# Synthesis, Characterization, and *In Vitro* and *In Silico* Studies of New Triazole Derivatives as Aromatase Inhibitors

**DOI:** 10.2174/0115734064316112240722092935

**Published:** 2024-09-18

**Authors:** Zeynep Livanur Üzmez, Derya Osmaniye, Yusuf Özkay, Zafer Asım Kaplancıklı

**Affiliations:** 1 Faculty of Pharmacy, Anadolu University, Eskişehir 26470, Turkey;; 2 Department of Pharmaceutical Chemistry, Faculty of Pharmacy, Anadolu University, Eskişehir 26470, Turkey;; 3 Central Analysis Laboratory, Faculty of Pharmacy, Anadolu University, Eskişehir 26470, Turkey

**Keywords:** Breast cancer, aromatase inhibitor, triazole, molecular docking, molecular dynamics, estrogen

## Abstract

**Introduction:**

Breast cancer is the most common type of cancer among women. Steroidal or non-steroidal aromatase inhibitors (NSAIs) are used clinically, and in most cancer diseases, resistance is the most important problem.

**Methods:**

The nitrogenous heterocyclic ring is noteworthy in the structure of non-steroidal aromatase inhibitors. This is the pharmacophore structure for aromatase inhibition. Because the enzyme interacts with the Fe^2+^ cation of the HEM structure in its active site, the most used agents in the clinic, such as anastrozole and letrozole, contain triazoles in their structures. Within the scope of this study, hybrid compounds containing both imidazole and triazole were synthesized.

**Results:**

The synthesis was carried out by a 4-step reaction. The anticancer effects of the compounds were evaluated by MTT assay performed on A549 and MCF-7 cancer cells. Compound **4d** showed anticancer activity against the MCF-7 cell line with IC_50_=6.7342 uM value. This compound exhibited anticancer activity against the A549 cell line with an IC_50_ = 17.1761 μM. In the MTT test performed on a healthy cell line to determine the cytotoxic effects of the compounds, the compound showed activity with a value of **4d** IC_50_=13.2088 uM. This indicates that the compound is not cytotoxic.

Additionally, BrdU analysis was performed to evaluate whether the compound inhibits DNA synthesis. These selective effects of the compounds on breast cancer strengthened their aromatase enzyme inhibitor potential. For this reason, experiments conducted with both *in vitro* and *in silico* methods revealed a compound with high aromatase inhibitor potential.

**Conclusion:**

The interactions observed as a result of molecular docking and dynamics studies are in harmony with activity studies. In particular, interactions with HEM600 demonstrate the activity potential of the compound.

## 1 INTRODUCTION

Death rates due to cancer, one of the most common causes of death worldwide, are increasing. Cancer, which affects many organs in the human body, can spread uncontrollably. Of the 12.7 million people diagnosed with cancer every year worldwide, 7.6 million die due to insufficient success in treatment. While lung, prostate, and colorectal cancers have the highest mortality rates in men, breast and colorectal cancers are among the leading causes of death in women [1].

Breast cancer, the most common form of malignant tumors, is the second leading cause of cancer-related deaths in women. Breast cancer poses a threat to many women of different age groups. It has been found that women diagnosed with breast cancer often have high estrogen levels [2, 3]. Among the different types of BC, the most common is the estrogen receptor-positive (ER+) subtype (70%) [4, 5]. Aromatase cytochrome P450 (CYP) is an enzyme that catalyzes the final rate-limiting step in estrogen biosynthesis. It supports estrogen synthesis. Suppressing estrogen levels is important for the treatment and control of hormone-dependent breast cancer [6]. CYP450 enzymes, consisting of 503 amino acids, also contain a HEM structure that carries Fe^2+^. The natural substrate of aromatase, which is involved in the coordination of the Fe^2+^-carrying heme group in the active site of the enzyme, is androstenedione [7]. For this reason, aromatase inhibitors (AIs) are among the most common hormonal drugs used in the treatment of breast cancer [8].

**Fig. (1):**
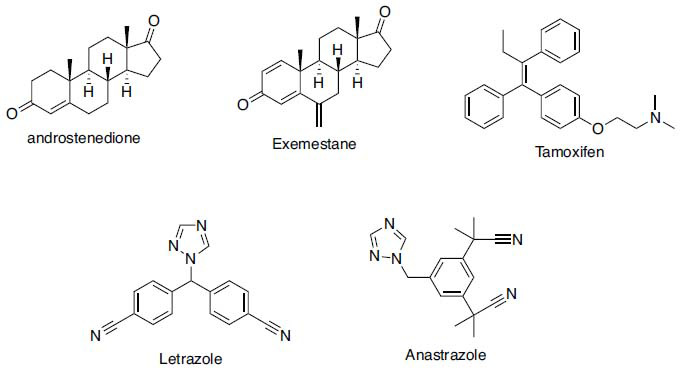
Clinically used anticancer drugs for estrogen-dependent breast cancer.

Tamoxifen (Fig. **1**), which has been the standard endocrine therapy in the treatment of estrogen-dependent breast cancer since the early 70s, acts as a selective estrogen receptor modulator. This drug has caused resistance and various side effects in the treatment of early and advanced breast cancer. Still, it has been quite effective in treatment [9]. AIs, which are generally divided into steroidal (exemestane, Fig. **1**) and non-steroidal (letrozole and anastrozole, Fig. **1**) based on their structure, suppress aromatase activity by interacting with the substrate binding site of aromatase [10].

When the structures of aromatase inhibitors are examined, it is seen that both 2^nd^ generation inhibitors (letrozole and anastrozole) contain a triazole ring. In addition, there are many compounds containing triazole rings with proven aromatase activity registered in the literature [9, 11-18]. For this purpose, within the scope of this study, 10 new triazole derivative compounds were synthesized, and their aromatase activities were investigated.

Letrozole is one of the most used aromatase inhibitors. When its structure is examined (Fig. **1**), two 4-cyanobenzene rings are seen connected to a triazole ring with a methylene bridge. The triazole ring is the pharmacophore structure for aromatase inhibition. The enzyme interacts with Fe^2+^ in its active site. That is why the triazole ring is kept constant when the structure of the compounds is designed. The imidazole ring is preferred over one of the phenyl rings in letrozole. The substituents of the phenyl ring also consist of electron-withdrawing substituents, including the cyano group.

## 2 MATERIALS AND METHODS

### 2.1 Chemistry

#### 2.1.1 General Information

The chemical substance and device information used are provided in the Supporting Information file.

##### 2.1.1.1 Method for the Synthesis of Target Compounds

The previously reported method was used to obtain the compounds 1-3 [19]. To obtain the target compounds (**4a-4j**), **compound 3** (0.001 mol, 0.3 g) was dissolved in acetone (10 mL). Potassium carbonate was added as a catalyst, and an equivalent amount of suitable 2-bromocetophenone derivatives was added to the obtained mixture. The resulting mixture was stirred at room temperature for 12 hours under a magnetic stirrer. After the end of the reaction, acetone was evaporated, and the remaining residue was washed with water. The obtained product was dried and crystallized from ethanol [19].

Compound **4a**

Yield: 88%, ^1^H-NMR (300 MHz, DMSO-*d_6_*): δ = 1.26 (3H, t, *J*=7.1 Hz, -CH_2_CH_3_), 2.39 (3H, s, -CH_3_), 2.42 (3H, s, -CH_3_), 4.37 (2H, q, *J*=7.0 Hz, -CH_2_CH_3_), 4.89 (2H, s, -CH_2_-), 7.36 (2H, d, *J*=8.0 Hz, 1,4-Disubstitutedbenzene), 7.76 (1H, s, imidazole), 7.92 (2H, d, *J*=8.2 Hz, 1,4-Disubstitutedbenzene). ^13^C-NMR (75 MHz, DMSO-*d_6_*): δ =10.97, 15.81, 21.66, 21.68, 41.09, 124.70, 128.56, 129.01, 129.82, 133.28, 135.14, 144.71, 148.44, 150.63, 193.34. HRMS (m/z): [M+H]^+^ calcd for C_17_H_19_N_5_OS: 342.1383; found: 342.1370.

Compound **4b**

Yield: 88%, ^1^H-NMR (300 MHz, DMSO-*d_6_*): δ = 1.26 (3H, t, *J*=7.1 Hz, -CH_2_CH_3_), 2.43 (3H, s, -CH_3_),3.85 (3H, s, -OCH_3_), 4.38 (2H, q, *J*=7.1 Hz, -CH_2_CH_3_), 4.86 (2H, s, -CH_2_-), 7.06 (2H, d, *J*=8.9 Hz, 1,4-Disubstitutedbenzene), 7.74 (1H, s, imidazole), 8.00 (2H, d, *J*=8.9 Hz, 1,4-Disubstitutedbenzene). ^13^C-NMR (75 MHz, DMSO-*d_6_*): δ =11.94, 13.57, 16.69, 40.97, 55.12, 113.45, 115.60, 128.63, 130.28, 132.41, 133.75, 136.52, 148.41, 164.05, 192.14. HRMS (m/z): [M+H]+ calcd for C_17_H_19_N_5_O_2_S: 358.1332; found: 358.1331.

Compound **4c**

Yield: 88%, ^1^H-NMR (300 MHz, DMSO-*d_6_*): δ = 1.26 (3H, t, *J*=7.1 Hz, -CH_2_CH_3_), 2.42 (3H, s, -CH_3_), 4.37 (2H, q, *J*=7.1 Hz, -CH_2_CH_3_), 4.94 (2H, s, -CH_2_-), 7.78 (1H, s, imidazole), 8.04 (2H, d, *J*=8.6 Hz, 1,4-Disubstitutedbenzene), 8.17 (2H, d, *J*=8.6 Hz, 1,4-Disubstitutedbenzene). ^13^C-NMR (75 MHz, DMSO-*d_6_*): δ =10.32, 14.98, 43.05, 116.35, 118.82, 128.57, 130.61, 132.35, 133.95, 136.71, 139.33, 148.20, 150.82, 193.58. HRMS (m/z): [M+H]+ calcd for C_17_H_16_N_6_OS: 353.1179; found: 353.1166.

Compound **4d**

Yield: 88%, ^1^H-NMR (300 MHz, DMSO-*d_6_*): δ = 1.26 (3H, t, *J*=7.1 Hz, -CH_2_CH_3_), 2.41 (3H, s, -CH_3_), 4.37 (2H, q, *J*=7.1 Hz, -CH_2_CH_3_), 4.97 (2H, s, -CH_2_-), 7.77 (1H, s, imidazole), 8.26 (2H, d, *J*=8.9 Hz, 1,4-Disubstitutedbenzene), 8.37 (2H, d, *J*=8.9 Hz, 1,4-Disubstitutedbenzene). ^13^C-NMR (75 MHz, DMSO-*d_6_*): δ = 11.82, 14.95, 16.64, 123.21, 124.74, 125.49, 129.27, 131.42, 133.80, 136.56, 140.57, 150.46, 193.29. HRMS (m/z): [M+H]+ calcd for C_16_H_16_N_6_O_3_S: 373.1077; found: 373.1071.

Compound **4e**

Yield: 88%, ^1^H-NMR (300 MHz, DMSO-*d_6_*): δ = 1.27 (3H, t, *J*=7.1 Hz, -CH_2_CH_3_), 2.42 (3H, s, -CH_3_), 4.38 (2H, q, *J*=7.1 Hz, -CH_2_CH_3_), 4.91 (2H, s, -CH_2_-), 7.39 (2H, t, *J*=8.9 Hz, 1,4-Disubstitutedbenzene), 7.75 (1H, s, imidazole), 8.12 (2H, dd, *J_1_*=5.5 Hz, *J_2_*=8.9 Hz, 1,4-Disubstitutedbenzene). ^13^C-NMR (75 MHz, DMSO-*d_6_*): δ =11.00, 11.04, 15.82, 40.81, 116.32 (d, *J*=22.1 Hz), 125.02, 128.44, 131.99 (d, *J*=9.5 Hz), 132.58 (d, *J*=2.8 Hz), 135.11, 148.21, 150.86, 165.74 (d, *J*=252.4 Hz), 192.53. HRMS (m/z): [M+H]+ calcd for C_16_H_16_N_5_OFS: 346.1132; found: 346.1125.

Compound **4f**

Yield: 88%, ^1^H-NMR (300 MHz, DMSO-*d_6_*): δ = 1.27 (3H, t, *J*=7.2 Hz, -CH_2_CH_3_), 2.42 (3H, s, -CH3), 4.37 (2H, q, J=7.3 Hz, -CH2CH3), 4.90 (2H, s, -CH2-), 7.63 (2H, t, J=8.3 Hz, 1,4-Disubstitutedbenzene), 7.78 (1H, s, imidazole), 8.04 (2H, d, J=8.5 Hz, 1,4-Disubstitutedbenzene). 13C-NMR (75 MHz, DMSO-d6): δ = 11.82, 14.93, 16.63, 42.83, 128.24, 128.31, 129.71, 130.54, 131.85, 131.95, 133.79, 134.53, 136.56, 193.36. HRMS (m/z): [M+H]+ calcd for C_16_H_16_N_5_OSCl: 362.0837; found: 362.0834.

Compound **4g**

Yield: 88%, ^1^H-NMR (300 MHz, DMSO-*d_6_*): δ = 1.26 (3H, t, *J*=7.1 Hz, -CH_2_CH_3_), 2.42 (3H, s, -CH_3_), 4.37 (2H, q, *J*=7.1 Hz, -CH_2_CH_3_), 4.90 (2H, s, -CH_2_-), 7.75-7.79 (3H, m, 1,4-Disubstitutedbenzene+imidazole), 7.96 (2H, d, *J*=8.6 Hz, 1,4-Disubstitutedbenzene). ^13^C-NMR (75 MHz, DMSO-*d_6_*): δ = 11.84, 14.95, 16.64, 42.81, 128.36, 129.87, 131.31, 132.03, 133.50, 133.85, 136.63, 148.29, 150.73, 193.22. HRMS (m/z): [M+H]+ calcd for C_16_H_16_N_5_OSBr: 406.0332; found: 406.0320.

Compound **4h**

Yield: 88%, ^1^H-NMR (300 MHz, DMSO-*d_6_*): δ = 1.25 (3H, t, *J*=7.1 Hz, -CH_2_CH_3_), 2.32 (3H, s, -CH_3_), 2.36 (3H, s, -CH_3_), 2.43 (3H, s, -CH_3_), 4.35 (2H, q, *J*=7.2 Hz, -CH_2_CH_3_), 4.78 (2H, s, -CH_2_-), 7.13-7.15 (2H, m, 1,2,4-Trisubstitutedbenzene), 7.72 (1H, s, imidazole), 7.83 (1H, d, *J*=7.7 Hz, 1,2,4-Trisubstitutedbenzene). ^13^C-NMR (75 MHz, DMSO-*d_6_*): δ =11.00, 15.79, 21.29, 21.37, 40.12, 42.92, 125.07, 126.84, 128.39, 130.14, 132.89, 133.59, 135.09, 138.62, 142.59, 158.36, 150.81, 196.56. HRMS (m/z): [M+H]+ calcd for C_18_H_21_N_5_OS: 356.1540; found: 356.1534.

Compound **4i**

Yield: 88%, ^1^H-NMR (300 MHz, DMSO-*d_6_*): δ = 1.26 (3H, t, *J*=7.0 Hz, -CH_2_CH_3_), 2.49 (3H, s, -CH_3_), 4.38 (2H, q, *J*=7.1 Hz, -CH_2_CH_3_), 4.79 (2H, d, *J*=2.4 Hz, -CH_2_-), 7.23-7.29 (1H, m, 1,2,4-Trisubstitutedbenzene), 7.44-7.51 (1H, m, 1,2,4-Trisubstitutedbenzene), 7.71 (1H, s, imidazole), 7.96-8.04 (1H, m, 1,2,4-Trisubstitutedbenzene). ^13^C-NMR (75 MHz, DMSO-*d_6_*): δ =11.01, 15.79, 40.17, 44.18 (d, *J*=7.5 Hz), 105.77 (t, *J*=26.8 Hz), 112.99 (dd, *J_1_*=2.5 Hz, *J_2_*=21.5 Hz), 121.38 (dd, *J_1_*=4.2 Hz, *J_2_*=12.6 Hz), 125.19, 128.37, 133.37 (dd, *J_1_*=3.4 Hz, *J_2_*=11.2 Hz), 135.13 (d, *J*=3.8 Hz), 148.09, 150.95, 162.43 (dd, *J_1_*=13.2 Hz, *J_2_*=255.2 Hz), 165.81 (dd, *J_1_*=12.6 Hz, *J_2_*=252.01 Hz), 190.42 (d, *J*=4.2 Hz). HRMS (m/z): [M+H]+ calcd for C_16_H_15_N_5_OF_2_S: 364.1038; found: 364.1020.

Compound **4j**

Yield: 88%, ^1^H-NMR (300 MHz, DMSO-*d_6_*): δ = 1.21 (3H, t, *J*=7.1 Hz, -CH_2_CH_3_), 2.39 (3H, s, -CH_3_), 4.24 (2H, q, *J*=7.1 Hz, -CH_2_CH_3_), 4.81 (2H, d, *J*=2.4 Hz, -CH_2_-), 7.59 (1H, dd, *J_1_*=2.0 Hz, *J_2_*=8.4 Hz, 1,2,4-Trisubstitutedbenzene), 7.76 (1H, d, *J*=2.0 Hz, 1,2,4-Trisubstitutedbenzene), 7.87 (1H, d, *J*=8.4 Hz, 1,2,4-Trisubstitutedbenzene), 8.15 (1H, s, imidazole). ^13^C-NMR (75 MHz, DMSO-*d_6_*): δ = 10.69, 15.54, 15.55, 42.97, 128.04, 129.53, 130.55, 131.96, 132.05, 135.37, 135.39, 135.79, 137.25, 148.99, 149.08, 194.93. HRMS (m/z): [M+H]+ calcd for C_16_H_15_N_5_OSCl_2_: 396.0447; found: 396.0430.

### 2.2 Anticancer Activity

#### 2.2.1 Cytotoxicity Studies

Cytotoxicity studies were carried out using the MTT method, based on the principle of forming purple formazan salt [20]. The MTT procedure was performed using a CO_2_ incubator during a 24-hour incubation period, as described in our previous publications. The results were detected with a microplate reader [21, 22].

#### 2.2.2 BrdU Analysis

Three different incubation periods were selected for the experiment, which was carried out according to the kit procedure. (BioVision, BrdU Cell Proliferation Assay Kit (Fluorometric)). Test results performed at 24-hour, 48-hour, and 72-hour periods were based on fluorimetric measurement of the color change caused by the addition of the stop solution. It enables us to find out where the anticancer effect of anticancer compounds originates from. In other words, the following question persists: by affecting which phase in the cell cycle is DNA synthesis inhibited? BrdU analysis is important to find the answer to this question.

#### 2.2.3 Aromatase Inhibition

In the experiment performed according to the kit procedure, IC_50_ values of the compound were calculated from the results obtained with the help of a microplate reader (BioVision, Aromatase (CYP19A) Inhibitor Screening Kit (Fluorometric)).

### 2.3 *In Silico* Studies

It was made by standard docking procedure using the Schrödinger program and 3EQM crystal (2.90 Å). Docking studies were implemented using standard arrangements of the Schrödinger Suite 2020 Update 2 program. Using the LigPrep 3.8 and Glide 7.1 interfaces, single precision (SP) placement was completed [23-26]. Molecular dynamics studies were performed, as previously reported, using the Schrodinger Desmond program. The POPE membrane model was used and studied at 310.55 K temperature. It was carried out by planning a time of 100 ns. Molecular dynamic (MD) simulations are considered an important computational tool for evaluating the time-dependent stability of a ligand in an active site for a drug-receptor complex. MD simulations for 100 ns were carried out to ensure the stability of the identified hits from the docking result. We performed the Desmond application using the standard force field (OPLS3e) of the Schrodinger Suite with a transferable intermolecular potential with a 3-point (TIP3P) water model followed by energy minimization of the complex. The neutralization of the system was achieved using Na^+^ and Cl^−^ ions to provide a final salt concentration of 0.15 M to simulate the physiological concentration of monovalent ions. Constant temperature (300K) and pressure (1.01325 bar) were employed with NPT (constant number of particles, pressure, and temperature) as an ensemble class. RESPA integrator was used to integrate the equations of motion. NH thermostats were used to keep the constant simulation temperature, and the MTK method was applied to control the pressure. Long-range electrostatic interactions were calculated by the pmE method. The cutoff for van der Waals and short-range electrostatic interactions was set at 9.0 Å. The equilibration of the system was performed with the default protocol provided in Desmond, which consists of a series of restrained minimizations and molecular dynamics simulations used to slowly relax the system. This procedure was also previously applied by our *in silico* study group. The MD simulation was performed using the above settings and following the completion of the system setup. Rg (radius of gyration), root mean square fluctuation (RMSF), and root mean square deviation (RMSD) values were calculated by the Desmond application [27-33].

## 3 RESULTS AND DISCUSSION

### 3.1 Chemistry

**Scheme 1:**
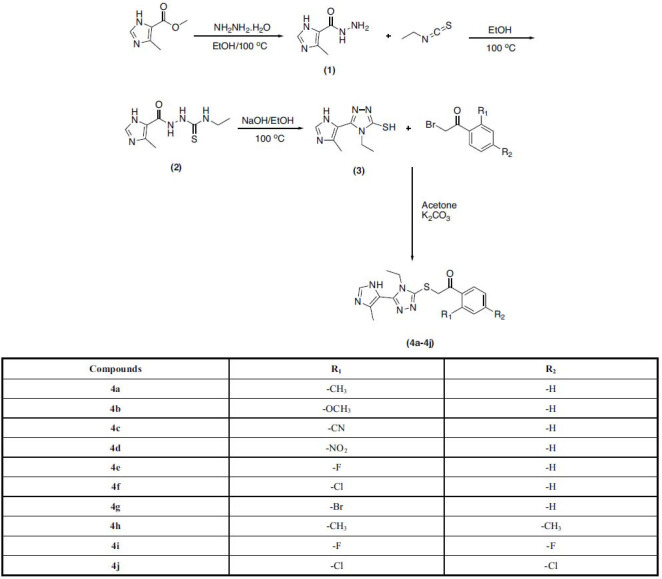
Synthesis pathway of compounds **4a-4j**.

The target compounds were obtained because of a 4-step synthesis reaction. The reaction steps are summarized in Scheme **1**. First, methyl 4-methyl-1*H*-imidazole-5-carboxylate was reacted with excess hydrazine hydrate at room temperature to obtain 4-methyl-1*H*-imidazole-5-carbohydrazide. *N*-Ethyl-2-(4-methyl-1*H*-imidazole-5-carbonyl) hydrazine-1-carbothioamide (compound **2**) was obtained by reacting the obtained hydrazide derivative (compound **1**) with ethyl isothiocyanate. Triazole ring closure reaction was performed by refluxing the thiourea derivative compound (compound **3**) in a basic medium. Finally, the obtained triazole (compound **3**) was reacted with 2-bromoacetophenone derivatives to reach the target compounds.

### 3.2 Biological Activity Study

#### 3.2.1 Cytotoxicity Studies

For activity studies, the 24-hour MTT procedure was applied. A549 (Lung cancer cell) (ATCC-CRM-CCL-185), MCF-7 (breast cancer cell) (ATCC-HTB-22), and NIH3T3 (normal fibroblast cell) (ATCC-CRL-1658) were used. The results obtained are presented in Table **1**. Looking at the activity results, compound **4d** is promising. Compound **4d** exhibited activity against the A549 cell line with an IC_50_=17.1761 µM. This compound exhibited activity against the MCF-7 cell line with IC_50_=6.7342 µM and showed cytotoxicity against the NIH3T3 cell line with IC_50_=13.2088 µM. The value shown against the MCF-7 cell line is especially important. Therefore, compound **4d** was subjected to the aromatase enzyme inhibition test *in vitro*. When the structure of compound **4d** was examined, unlike other compounds, it carried a nitro substitution in the fourth position of the phenyl ring. The compound (**4g**), which has a bromine substituent in the fourth position except for the nitro substituent, exhibited good activity, especially on the A549 cell line. However, the fact that it kills more healthy cells shows that bromine is cytotoxic.

#### 3.2.2 Brdu Analysis

With the Brdu studies, the potential of compound **4d** to inhibit DNA synthesis was examined in 24-, 48- and 72-hour periods. The results obtained are presented in Fig. (**2**). For the 24-hour incubation period, DNA synthesis inhibition was achieved by 16.1% at the IC_50_/2 dose, 28.1% at the IC_50_ dose, and 63.3% at the 2xIC_50_ dose. For the 48-hour incubation period, DNA synthesis inhibition was achieved by 40.4% at IC_50_/2 dose, 77.5% at IC_50_ dose, and 79.0% at 2xIC_50_ dose. For the 72-hour incubation period, DNA synthesis inhibition was achieved by 77.8% at IC_50_/2 dose, 80.6% at IC_50_ dose, and 92.4% at 2xIC_50_ dose. It shows that these compounds show anticancer activity by inhibiting DNA synthesis. Since DNA synthesis occurs in the G2 phase of the cell cycle, it would be a correct approach to say that the compounds affect the G2 phase.

#### 3.2.3 Aromatase Inhibition

**Fig. (3):**
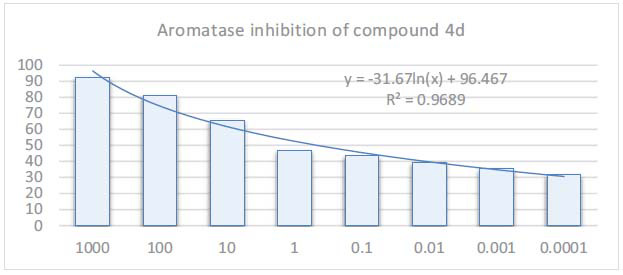
Aromatase inhibition diagram of compound **4d**.

According to the results obtained, it showed aromatase enzyme inhibition with IC_50_=0.097 µM (Fig. **3**). Unlike other compounds, this compound has a **4d** nitro substituent. Many triazole derivatives have previously been synthesized by the authors, and their activity potentials have been examined. A summary of these compounds is presented in Fig. (**4**). The aromatic ring attached to the triazole ring was changed, and it was aimed to reach more promising derivatives. Molecule **1** had a 2,4-dichlorophenoxy structure attached to the triazole ring. Molecule-**2** had a pyrimidine-thionyl structure. Molecule **4d** had a 4-methylimidazole structure. Molecule-**2** had the highest activity when evaluated on MCF-7 cells. The heteroaromatic ring was thought to increase activity. It appeared that the heteroatom bridge attached to the triazole ring contributed significantly to aromatase activity.

### 3.3 *In Silico* Study

#### 3.3.1 Molecular Docking Study

Molecular docking studies were performed using 3EQM crystal. The docking scores of compounds **4a-4i** were -4.419, -4.316, -3.396, -3.112, -4.922, -4.412, -4.587, -4.988, -4.222 kcal/mol, respectively. Compound **4j** did not fit into the enzyme active site. Fig. (**5**) presents the interactions of both letrozole, known as an aromatase inhibitor, and compound **4d** in the enzyme active site. Compound **4d** interacted with HEM600 with both the triazole and imidazole ring (double pi-pi interaction). In letrozole, the interaction with HEM600 was provided by the triazole ring (pi-pi interaction and pi-cation interaction) (3.32 Å and 3.32 Å). A salt bridge (4.39 Å) was formed between the nitro group of compound **4d** and the hydroxy group of Asp309. Also, the phenyl group had formed an aromatic hydrogen bond with the carbonyl group of Leu477.

#### 3.3.2 Molecular Dynamic Study

Instant images were obtained because of molecular docking studies. However, the persistence of these images was more accurate to prove activity. For this reason, molecular dynamics studies were carried out with compound **4d**, which was determined as the active compound because of activity and molecular docking studies. POPE was used as the membrane type in the dynamics studies performed for 100 ns. The temperature was carried out at 310.55 K. RMSD and RMSF parameters were obtained because the studies showed stability. The RMSD parameter was expected to be below 3A. In the resulting graph (Fig. **6A**), RMSD increased to 1.75-2.00 A up to 5ns and remained constant in this range for 100 ns. This demonstrates the stability of the compound in the enzyme active site.

According to the RMSF results (Fig. **6B**), interacting amino acids and distances can be listed as follows: Gly69 (0.82 Å), Gly72 (0.68 Å), Arg115 (0.44 Å), Ile133 (0.45 Å), Phe221 (0.62 Å), Trp224 (0.65 Å), Gln225 (0.63 Å), Leu228 (0.58 Å), Leu304 (0.70 Å), Ala307 (0.59 Å), Thr310 (0.61 Å), Val369 (0.60 Å), Val370 (0.57 Å), Asp371 (0.55 Å), Met374 (0.45 Å), Cys437 (0.41 Å), His475 (0.76 Å), Leu477 (0.60 Å), and Ser478 (0.56 Å).

According to the video obtained because of the dynamic and Fig. (**6C**), the list of interacting amino acids is listed in Table **2**. The continuous interaction (Fig. **6D**) with Met374 was remarkable. This amino acid interacts with the cyano group of letrozole. It is a very important amino acid in terms of both activity and stability. For compound **4d,** the carbonyl group provided this interaction. Although the nitro group itself does not interact, it may have strengthened this interaction thanks to the conformation in which it holds the carbonyl. Therefore, the carbonyl group from the 2-bromoaceto-phenone residue contributed greatly to the activity.

Fig. (**6D**) shows the amino acid interactions with time. The uninterrupted interaction with Met374, an essential amino acid for aromatase enzyme inhibition, is striking here. Fig. (**6E**) presents over 40% of interactions. From here, it is seen that the interaction with Met374 is provided by the carbonyl group

## CONCLUSION

Within the scope of this study, 10 new triazole derivative compounds that could be aromatase inhibitors were synthesized. As in the reference drug letrozole, the triple aromatic ring structure was attempted to be preserved. Two of these rings, the azole group (imidazole and triazole), were chosen to observe their contribution to the activity. As a result of adhesive biological activity studies, compound **4d** came to the forefront. The difference between the compound and the compounds previously synthesized by our team (Fig. **3**) is that it contains imidazole as the third aromatic structure. This ring system showed weaker aromatase inhibition than the 2,4-dichlorophenyl ring. Compared to the pyrimidine ring, aromatase inhibition is observed at similar levels, although weaker. This shows that the fact that the third ring is a 6-membered ring makes a positive contribution to the activity. A phenyl ring with halogen substituents relative to a heteroaromatic ring enhances aromatase inhibition.

**Fig. (5):**
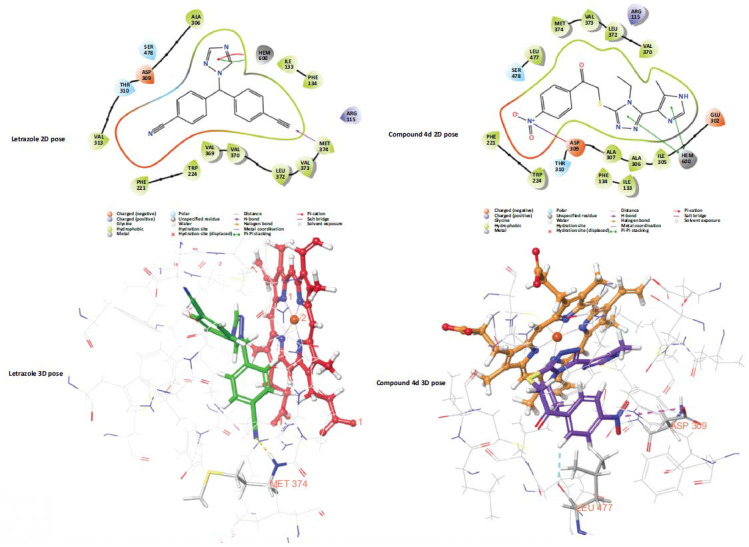
Docking poses of letrozole and compound **4d** with aromatase enzyme (PDB ID:3EQM). Two-dimensional poses for top-row letrozole and compound **4d**. Bottom row Two-dimensional poses for letrozole and compound **4d**.

**Fig. (6):**
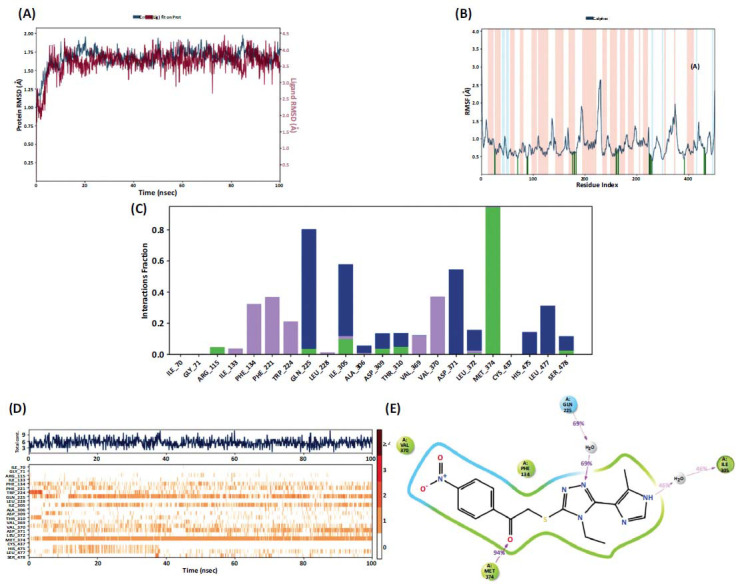
(**A-E**) Dynamic simulation results of compound **4d** with aromatase enzyme (PDB ID:3EQM).

Dynamic studies performed with compound 4d show that the compound is located close to HEM in the enzyme active site. Additionally, dynamic studies proved that the compound is stable in the enzyme active site. The continuous interaction with Met374, an important amino acid for the enzyme active site (Fig. **5D**), once again demonstrated the strong inhibition profile. As a result, 2-((4-ethyl-5-(5-methyl-1*H*-imidazol-4-yl)-4*H*-1,2,4-triazol-3-yl) thio)-1- was obtained within the scope of this study. The compound (4-nitrophenyl) ethan-1-one showed promising activity. The nitro group in the structure of this compound brought the carbonyl group into a conformation that could interact with Met374. Compared to previous studies, it appears that the imidazole ring does not contribute any extra activity. At the same time, the absence of the ether or thioether bridge in this study, which was present in previous studies (Fig. **3**), may have negatively affected the activity. In future studies, ether or thioether bridges will be added to compounds that will be developed based on this structure. In this way, the substituents affecting the activity will be tried to be understood more clearly.

## Figures and Tables

**Fig. (2):**
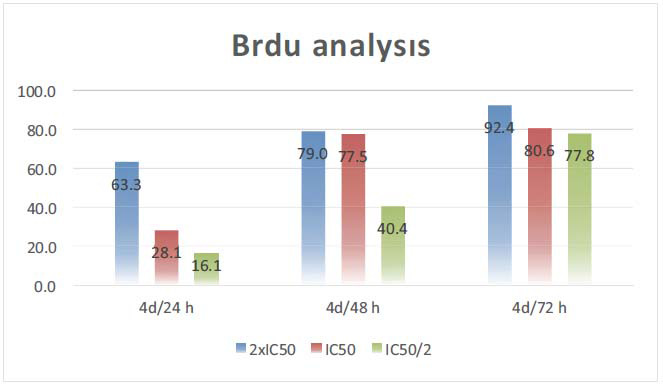
BRDU analysis results of compound **4d**.

**Fig. (4):**
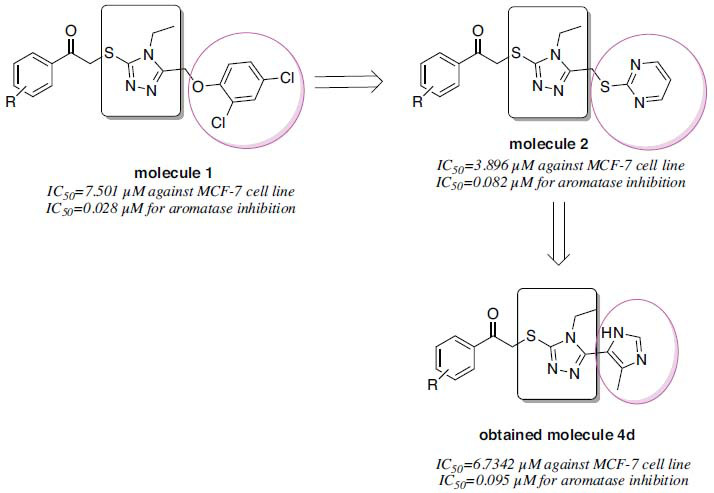
Comparison of previously obtained triazole derivatives.

**Table 1:** Anticancer activity results of obtained compounds 4a-4j against A549, MCF-7 and NIH3T3 cell lines (IC_50_= µM).

**Compounds**	**A549**	**MCF-7**	**NIH3T3**
**4a**	66.5279	34.8965	38.0379
**4b**	>100	31.1515	71.5025
**4c**	77.6881	>100	62.3366
**4d**	17.1761	6.7342	13.2088
**4e**	54.3087	86.8249	41.8471
**4f**	29.7958	37.7931	16.4305
**4g**	14.4195	25.2325	13.8353
**4h**	17.1700	>100	27.2419
**4i**	86.2278	64.4496	27.2409
**4j**	66.0598	28.9076	18.4245

**Note:** ^a^ The test results were expressed as means of triplicate assays ± SD.

**Table 2:** Interactions for compound 4d with the aromatase enzyme (PDB ID:3EQM).

**Type of Interactions**	**Amino Acids**
Hydrogen bond	Arg115, Gln225, Ile305, Asp309, Thr310, Leu372, Met374, Ser478
Water-mediated hydrogen bonding	Gln225, Ile305, Ala306, Asp309, Thr310, Asp371, Leu372, His475, Leu477, Ser478
Hydrophobic interactions	Ile133, Phe134, Phe221, Trp224, Leu228, Ile305, Ala306, Val369, Val370, Leu372
Aromatic hydrogen bond	Leu372, Phe221, Leu477, Trp224, Asp309

## Data Availability

The data and supportive information are available within the article.

## LIST OF ABBREVIATIONS

NSAIs: Non-steroidal Aromatase Inhibitors
CYP: Cytochrome P450
AIs: Aromatase Inhibitors
SP: Single Precision
MD: Molecular Dynamic
TIP3P: Transferable Intermolecular Potential with a 3-point
RMSD: Root Mean Square Deviation
RMSF: Root Mean Square Fluctuation
