# Supplementary material for: Synthesis, Characterization, and In Vitro and In Silico Studies of New Triazole Derivatives as Aromatase Inhibitors
Source: Med Chem. 2024 Sep 18;21(4):309–18. doi: 10.2174/0115734064316112240722092935 (PMC12606616; doi:10.2174/0115734064316112240722092935)
Supplement: Supplementary file 1 — Supplementary material is available on the publisher's website along with the published article. [file MC-21-4-309_SD1.pdf]

## Supplementary Materials

# Synthesis, Characterization, and *In Vitro* and *In Silico* Studies of New Triazole Derivatives as Aromatase Inhibitors

Zeynep Livanur Üzmez[1*], Derya Osmaniye[2,3*], Yusuf Özkay[2,3] and Zafer Asım Kaplancıklı[2]

*[1]Faculty of Pharmacy, Anadolu University, Eskişehir 26470, Turkey; [2]Department of Pharmaceutical Chemistry, Faculty of Pharmacy, Anadolu University, Eskişehir 26470, Turkey; [3]Central Analysis Laboratory, Faculty of Pharmacy, Anadolu University, Eskişehir 26470, Turkey*

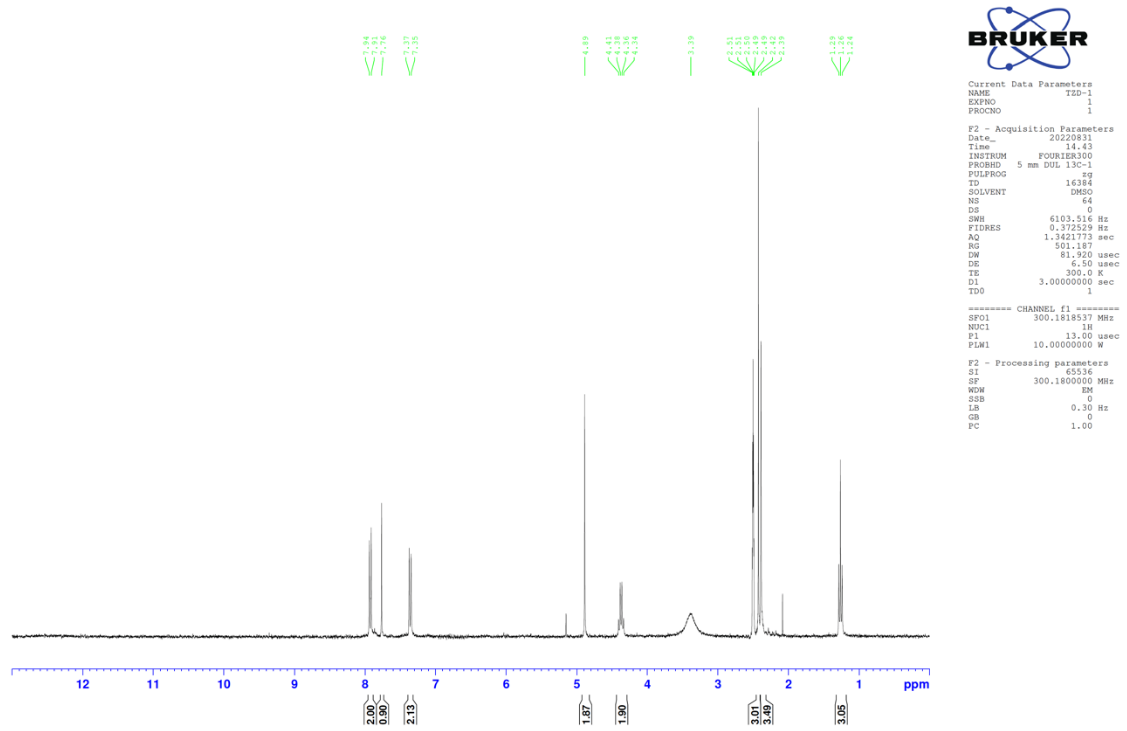


**Spectra 1.** $^{1}$H-NMR spectra of compound **4a**

 © 2025 Bentham Science Publishers

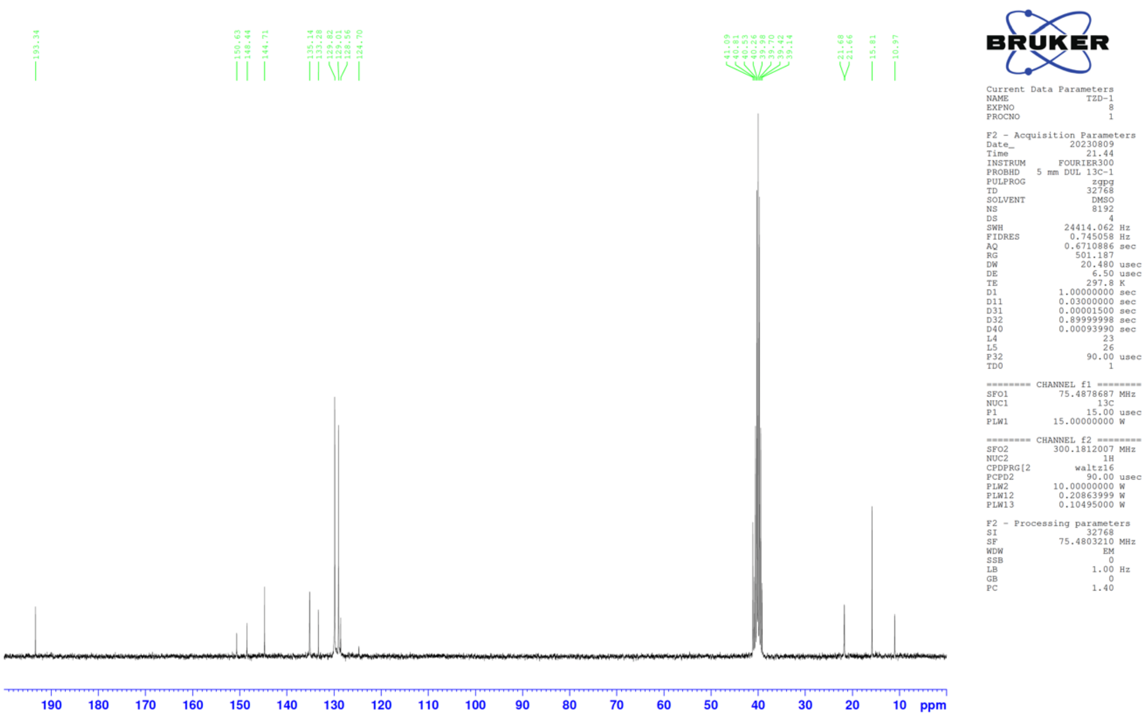


**Spectra 2.** $^{13}$C-NMR spectra of compound **4a**

Formula Predictor Report - TZD-1_27.lcd Page 1 of 1

Data File: C:\LabSolutions\Data\Analiz\derya\TZD-1_27.lcd

| Elmt | Val. | Min | Max | Elmt | Val. | Min | Max | Elmt | Val. | Min | Max | Use Adduct |
|---|---|---|---|---|---|---|---|---|---|---|---|---|
| H | 1 | 15 | 35 | F | 1 | 0 | 0 | Br | 1 | 0 | 0 | H |
| C | 4 | 17 | 35 | P | 3 | 0 | 0 | Ru | 2 | 0 | 0 | Na |
| N | 3 | 5 | 5 | S | 2 | 0 | 2 | Pd | 2 | 0 | 0 | K |
| O | 2 | 0 | 2 | Cl | 1 | 0 | 0 | I | 3 | 0 | 0 | NH4 |

| | | | | | |
|---|---|---|---|---|---|
| Error Margin (ppm): | 5 | DBE Range: | 5.0 - 20.0 | Electron Ions: | both |
| HC Ratio: | unlimited | Apply N Rule: | yes | Use MSn Info: | yes |
| Max Isotopes: | 3 | Isotope RI (%): | 1.00 | Isotope Res: | 9000 |
| MSn Iso RI (%): | 10.00 | MSn Logic Mode: | AND | Max Results: | 50 |

Event#: 1 MS(E+) Ret. Time : 2.147 Scan# : 323

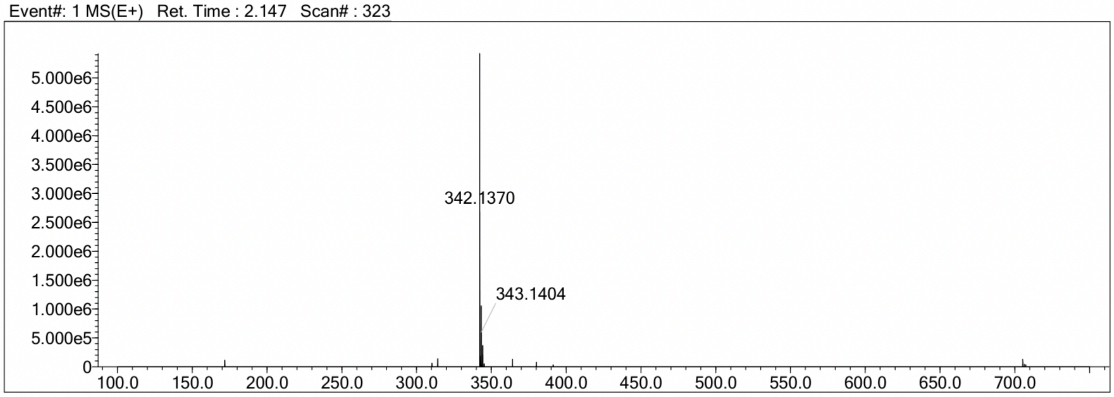


Measured region for 342.1370 m/z

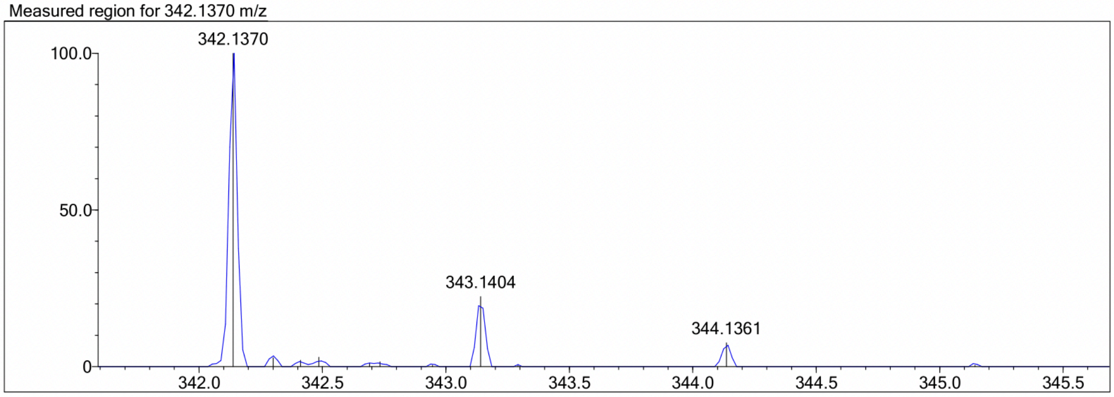


C17 H19 N5 O S [M+H]+ : Predicted region for 342.1383 m/z

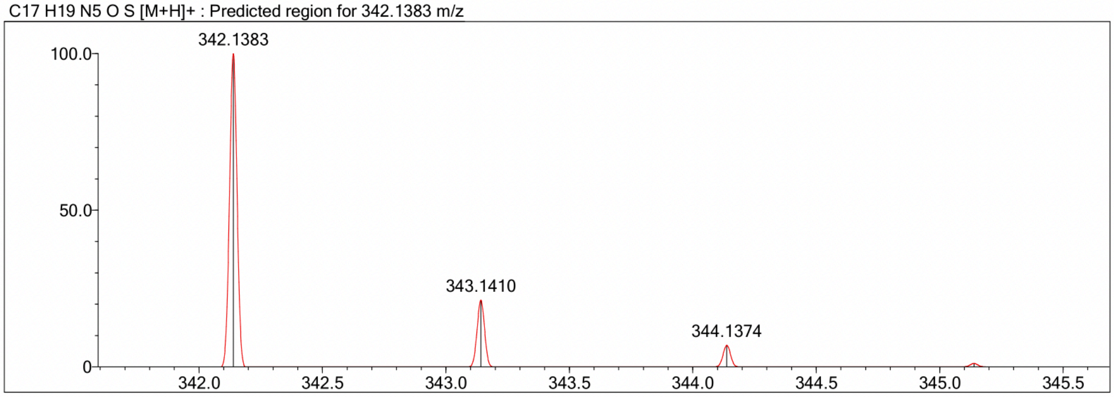


| Rank | Score | Formula (M) | Ion | Meas. m/z | Pred. m/z | Df. (mDa) | Df. (ppm) | Iso | DBE |
|---|---|---|---|---|---|---|---|---|---|
| 1 | 82.30 | C17 H19 N5 O S | [M+H]+ | 342.1370 | 342.1383 | -1.3 | -3.80 | 88.50 | 11.0 |

**Spectra 3.** HRMS spectra of compound **4a**

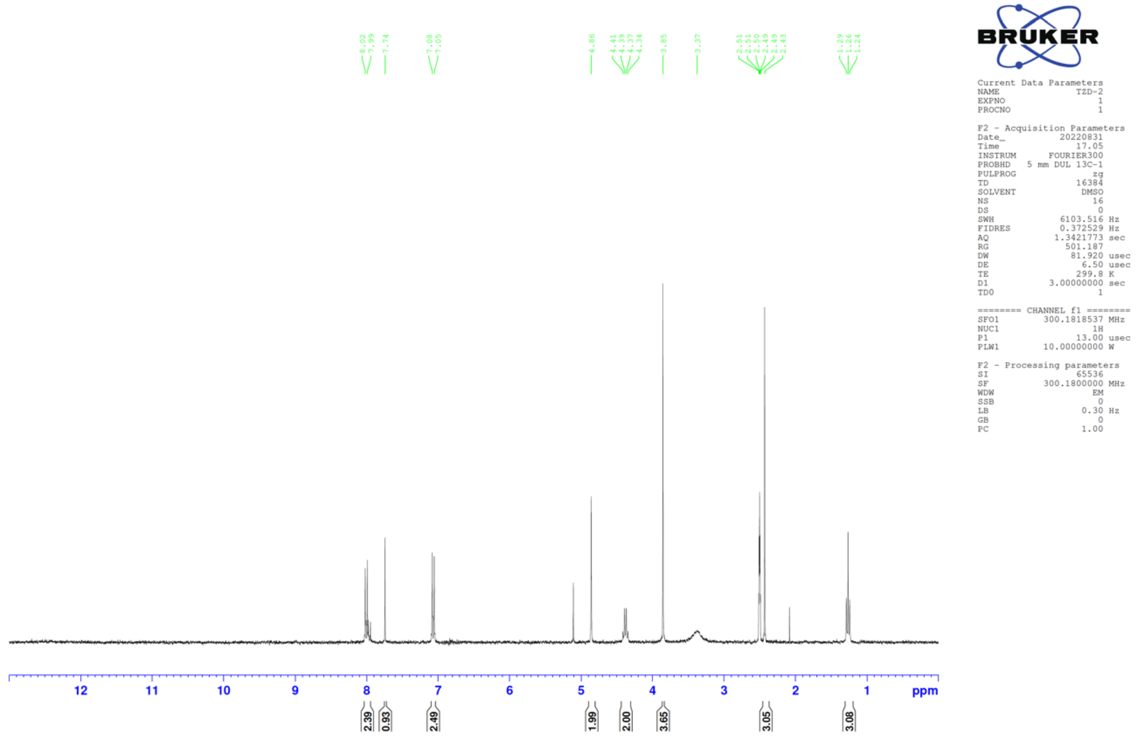

**Spectra 4.** $^{1}$H-NMR spectra of compound **4b**

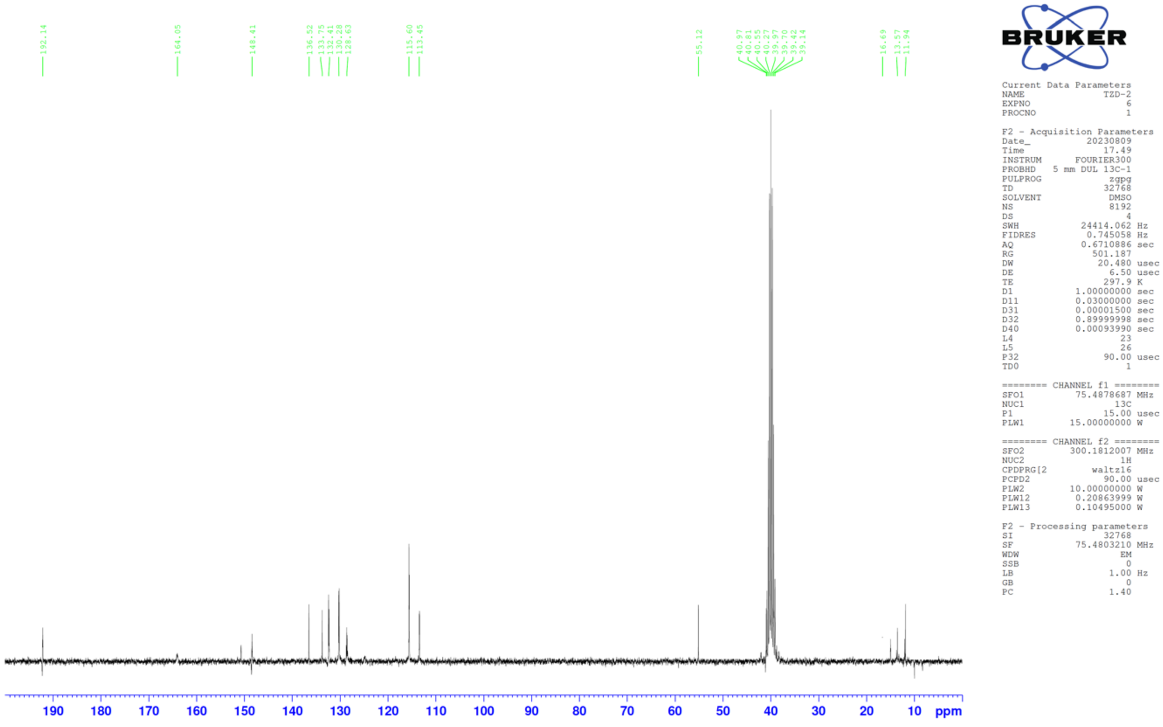

**Spectra 5.** $^{13}$C-NMR spectra of compound **4b**

Formula Predictor Report - T2D-2_235.lcd Page 1 of 1

Data File: C:\LabSolutions\Data\Analiz\derya\T2D-2_235.lcd

| Elmt | Val. | Min | Max | Elmt | Val. | Min | Max | Elmt | Val. | Min | Max | Use Adduct |
|---|---|---|---|---|---|---|---|---|---|---|---|---|
| H | 1 | 5 | 35 | F | 1 | 0 | 0 | Br | 1 | 0 | 0 | H |
| C | 4 | 5 | 35 | P | 3 | 0 | 0 | Ru | 2 | 0 | 0 | Na |
| N | 3 | 5 | 7 | S | 2 | 1 | 1 | Pd | 2 | 0 | 0 | K |
| O | 2 | 0 | 2 | Cl | 1 | 0 | 0 | I | 3 | 0 | 0 | NH4 |

| | | | | | |
|---|---|---|---|---|---|
| Error Margin (ppm): | 5 | DBE Range: | 5.0 - 20.0 | Electron Ions: | both |
| HC Ratio: | unlimited | Apply N Rule: | yes | Use MSn Info: | yes |
| Max Isotopes: | 3 | Isotope RI (%): | 1.00 | Isotope Res: | 9000 |
| MSn Iso RI (%): | 10.00 | MSn Logic Mode: | AND | Max Results: | 50 |

Event#: 1 MS(E+) Ret. Time : 2.227 Scan# : 335

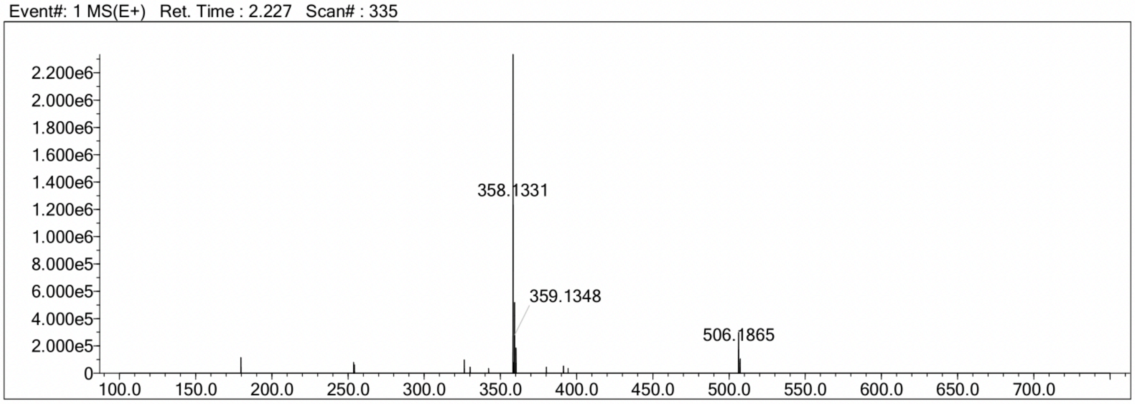


Measured region for 358.1331 m/z

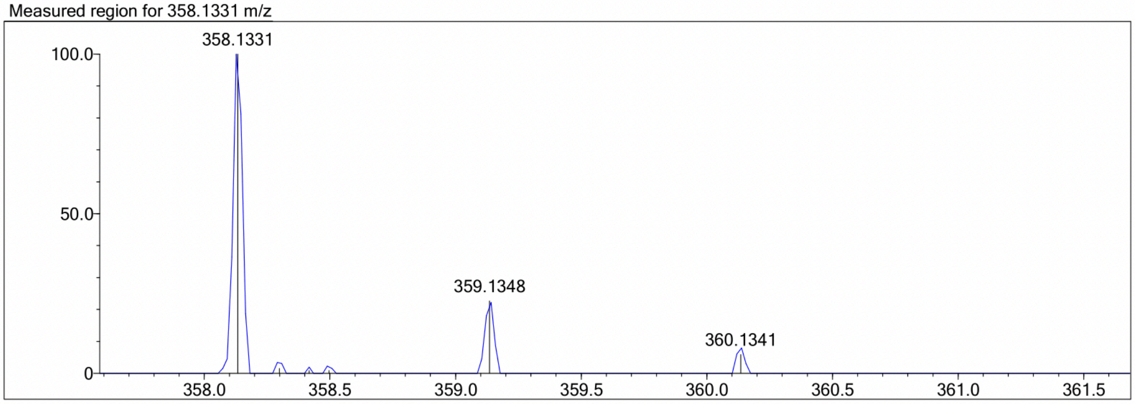


C17 H19 N5 O2 S [M+H]+ : Predicted region for 358.1332 m/z

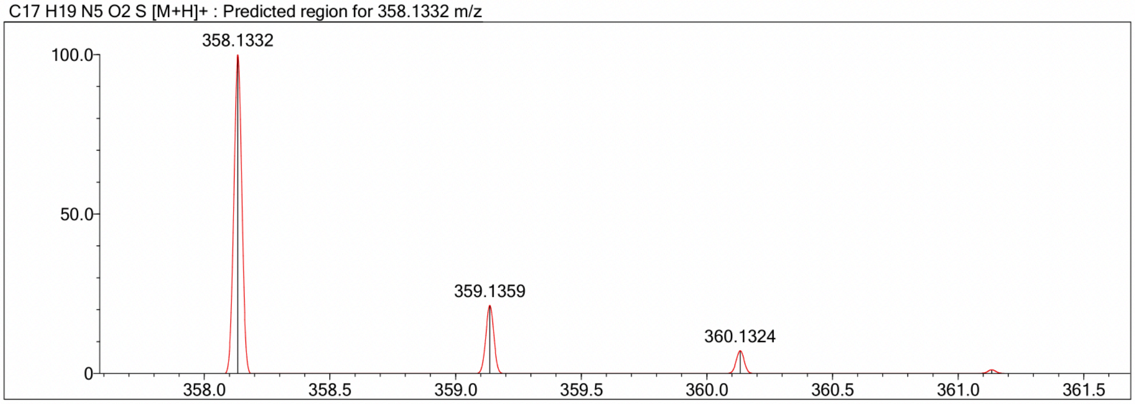


| Rank | Score | Formula (M) | Ion | Meas. m/z | Pred. m/z | Df. (mDa) | Df. (ppm) | Iso | DBE |
|---|---|---|---|---|---|---|---|---|---|
| 1 | 81.29 | C17 H19 N5 O2 S | [M+H]+ | 358.1331 | 358.1332 | -0.1 | -0.28 | 81.29 | 11.0 |

**Spectra 6.** HRMS spectra of compound **4b**

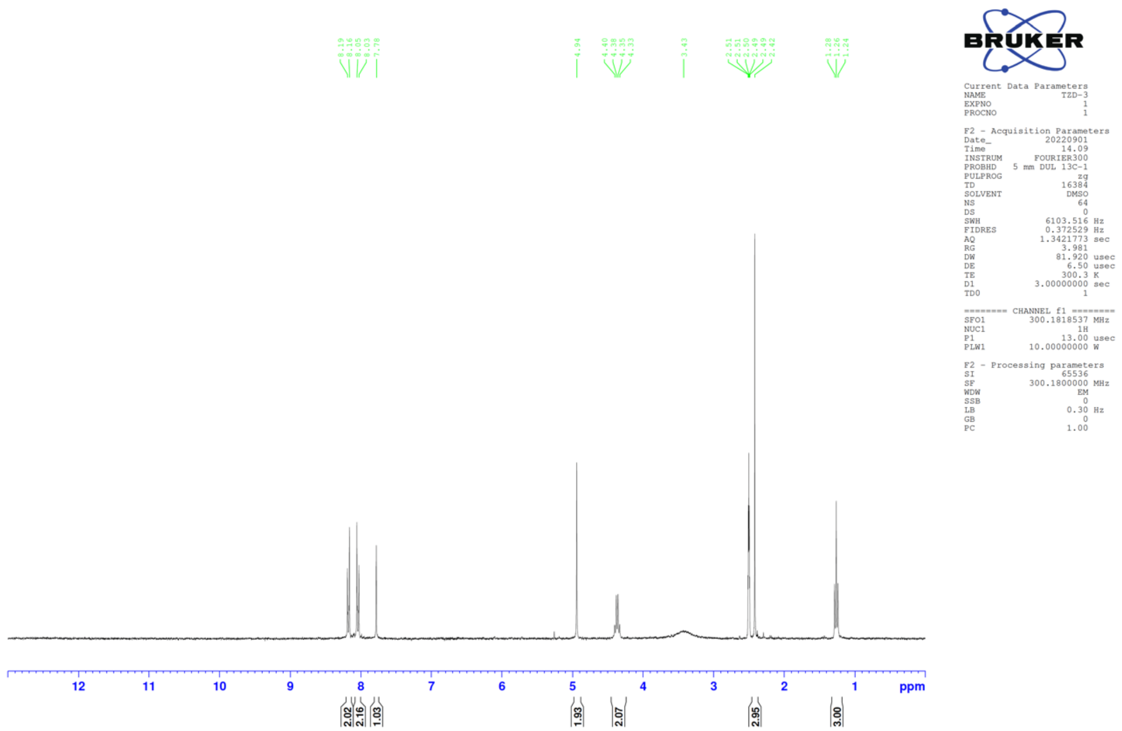


**Spectra 7.** $^{1}$H-NMR spectra of compound **4c**

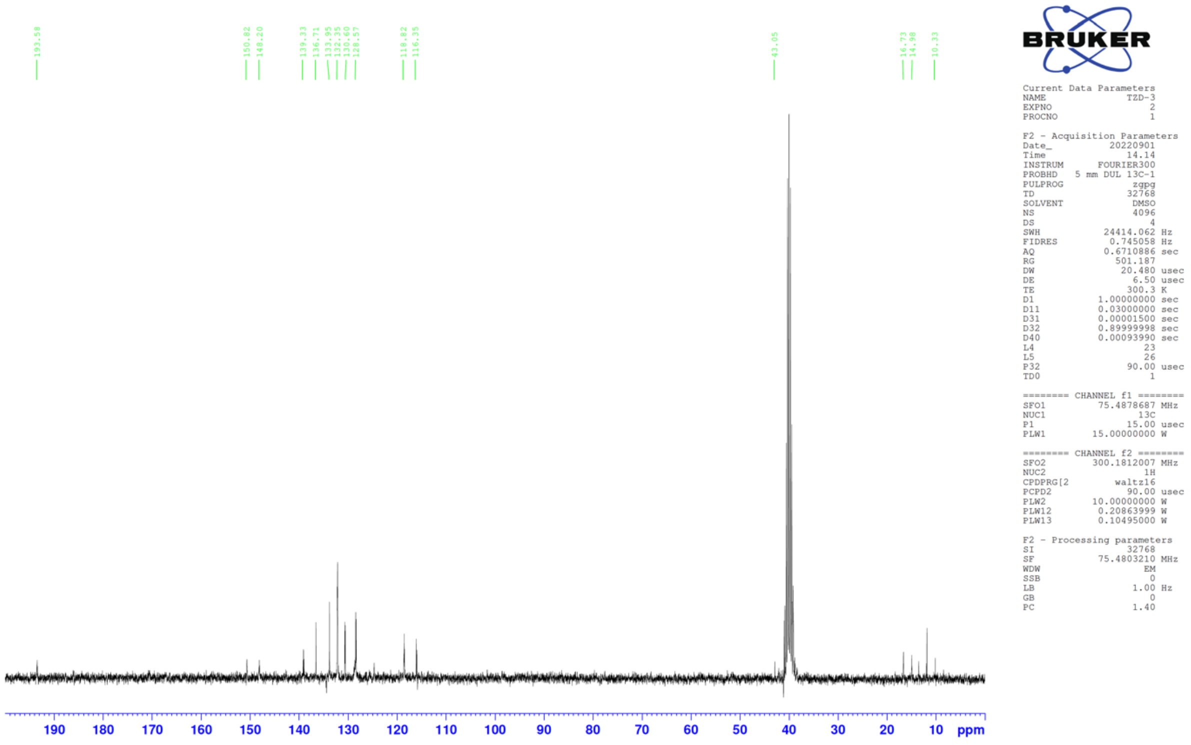


**Spectra 8.** $^{13}$C-NMR spectra of compound **4c**

Formula Predictor Report - T2D-3_236.lcd Page 1 of 1

Data File: C:\LabSolutions\Data\Analiz\derya\T2D-3_236.lcd

| Elmt | Val. | Min | Max | Elmt | Val. | Min | Max | Elmt | Val. | Min | Max | Use Adduct |
|---|---|---|---|---|---|---|---|---|---|---|---|---|
| H | 1 | 16 | 35 | F | 1 | 0 | 0 | Br | 1 | 0 | 0 | H |
| C | 4 | 17 | 35 | P | 3 | 0 | 0 | Ru | 2 | 0 | 0 | Na |
| N | 3 | 6 | 6 | S | 2 | 1 | 1 | Pd | 2 | 0 | 0 | K |
| O | 2 | 0 | 1 | Cl | 1 | 0 | 0 | I | 3 | 0 | 0 | NH4 |

| | | | | | |
|---|---|---|---|---|---|
| Error Margin (ppm): | 5 | DBE Range: | 5.0 - 20.0 | Electron Ions: | both |
| HC Ratio: | unlimited | Apply N Rule: | yes | Use MSn Info: | yes |
| Max Isotopes: | 3 | Isotope RI (%): | 1.00 | Isotope Res: | 9000 |
| MSn Iso RI (%): | 10.00 | MSn Logic Mode: | AND | Max Results: | 50 |

Event#: 1 MS(E+) Ret. Time : 2.040 Scan# : 307

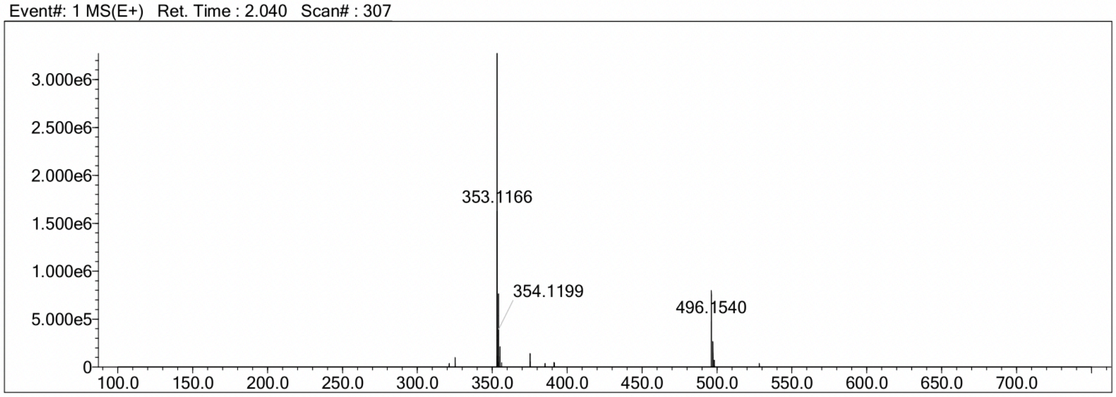


Measured region for 353.1166 m/z

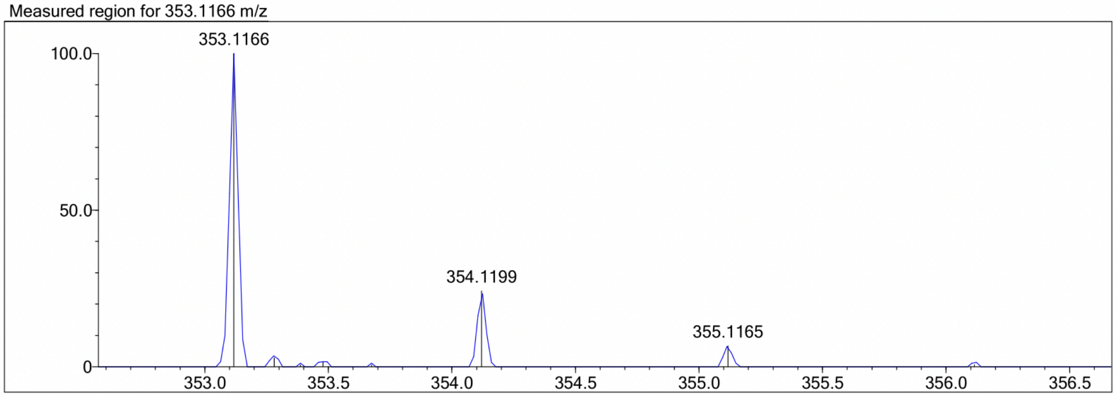


C17 H16 N6 O S [M+H]+ : Predicted region for 353.1179 m/z

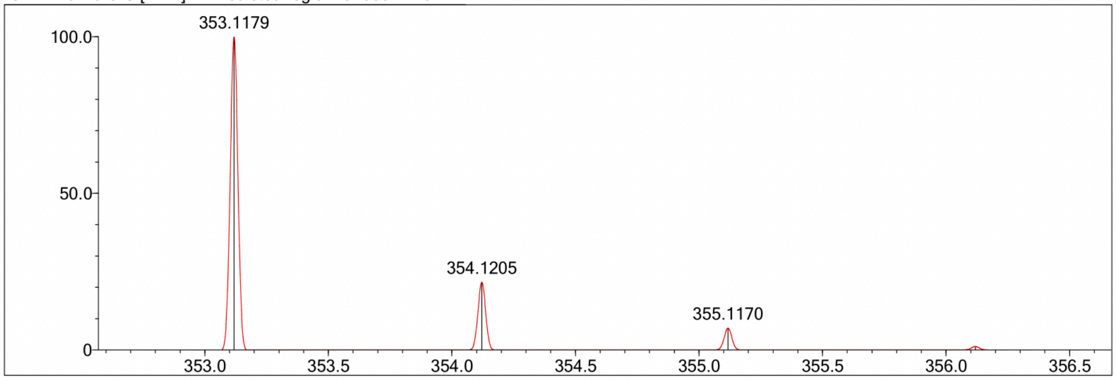


| Rank | Score | Formula (M) | Ion | Meas. m/z | Pred. m/z | Df. (mDa) | Df. (ppm) | Iso | DBE |
|---|---|---|---|---|---|---|---|---|---|
| 1 | 92.98 | C17 H16 N6 O S | [M+H]+ | 353.1166 | 353.1179 | -1.3 | -3.68 | 99.66 | 13.0 |

**Spectra 9.** HRMS spectra of compound **4c**

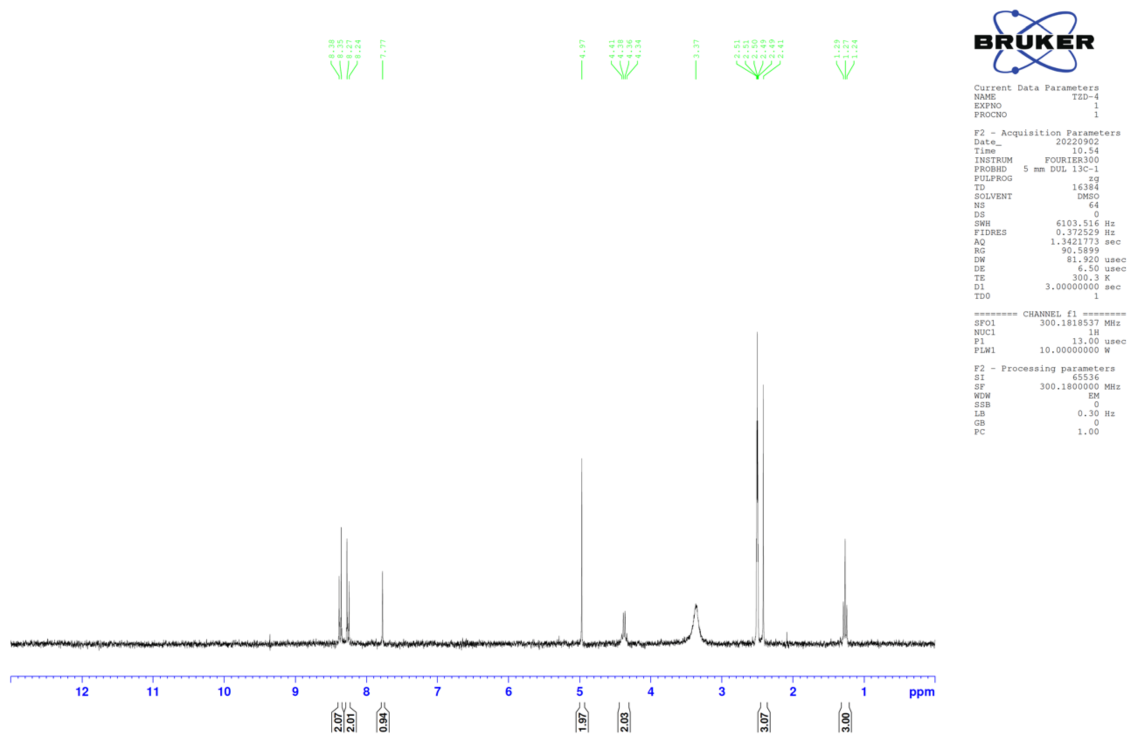

**Spectra 10.** $^{1}$H-NMR spectra of compound **4d**

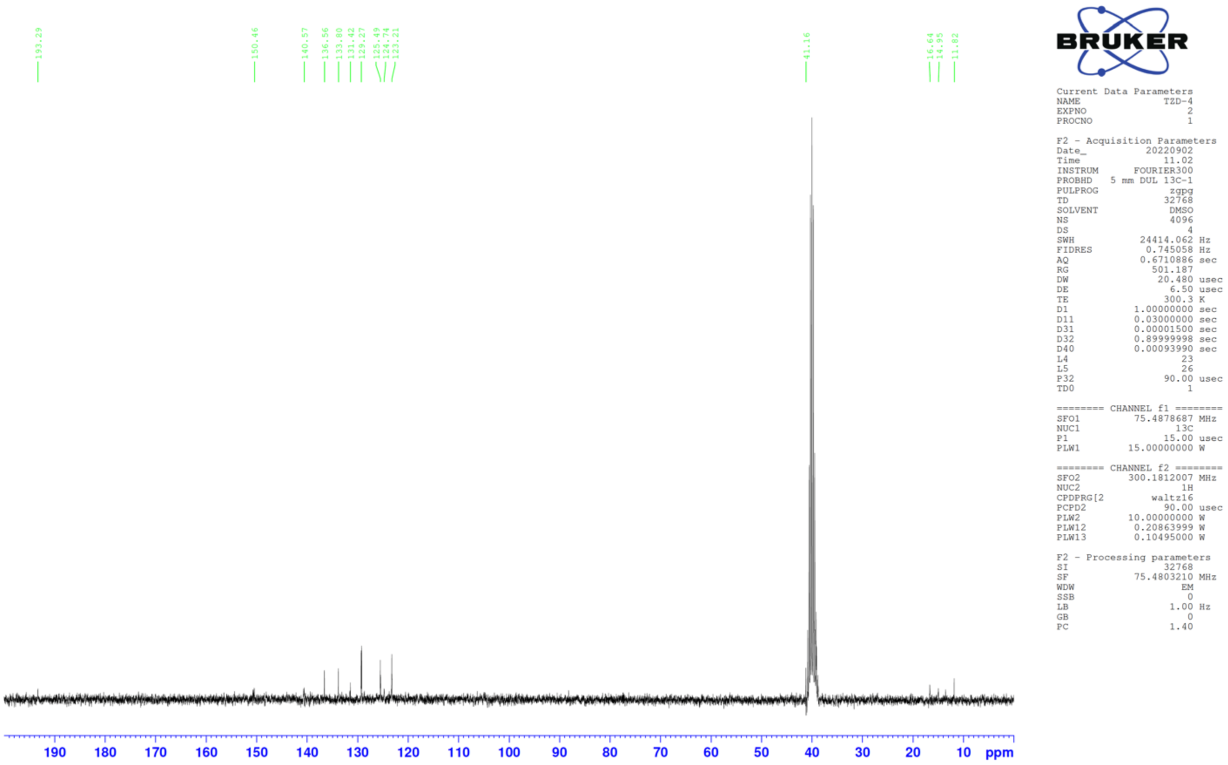

**Spectra 11.** $^{13}$C-NMR spectra of compound **4d**

Formula Predictor Report - T2D-4_237.lcd Page 1 of 1

Data File: C:\LabSolutions\Data\Analiz\derya\T2D-4_237.lcd

| Elmt | Val. | Min | Max | Elmt | Val. | Min | Max | Elmt | Val. | Min | Max | Use Adduct |
|---|---|---|---|---|---|---|---|---|---|---|---|---|
| H | 1 | 16 | 35 | F | 1 | 0 | 0 | Br | 1 | 0 | 0 | H |
| C | 4 | 16 | 35 | P | 3 | 0 | 0 | Ru | 2 | 0 | 0 | Na |
| N | 3 | 6 | 6 | S | 2 | 1 | 1 | Pd | 2 | 0 | 0 | K |
| O | 2 | 0 | 3 | Cl | 1 | 0 | 0 | I | 3 | 0 | 0 | NH4 |

| | | | | | |
|---|---|---|---|---|---|
| Error Margin (ppm): | 5 | DBE Range: | 5.0 - 20.0 | Electron Ions: | both |
| HC Ratio: | unlimited | Apply N Rule: | yes | Use MSn Info: | yes |
| Max Isotopes: | 3 | Isotope RI (%): | 1.00 | Isotope Res: | 9000 |
| MSn Iso RI (%): | 10.00 | MSn Logic Mode: | AND | Max Results: | 50 |

Event#: 1 MS(E+) Ret. Time : 2.067 Scan# : 311

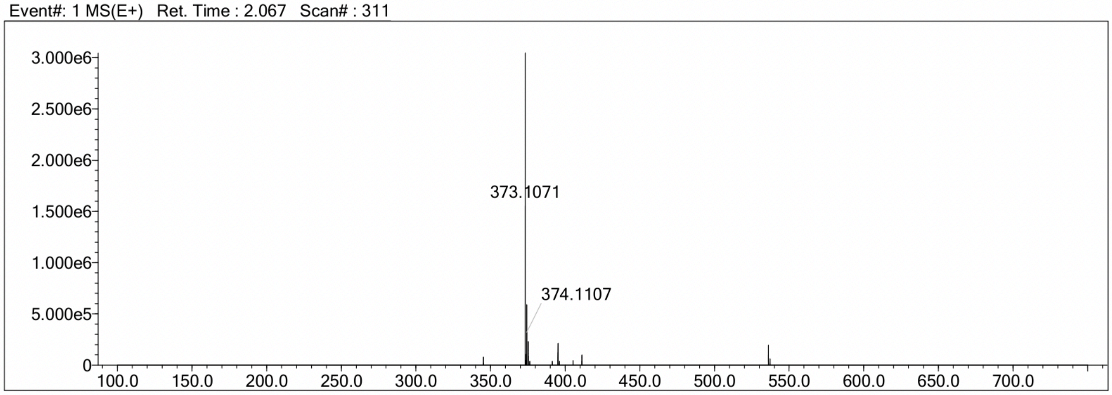


Measured region for 373.1071 m/z

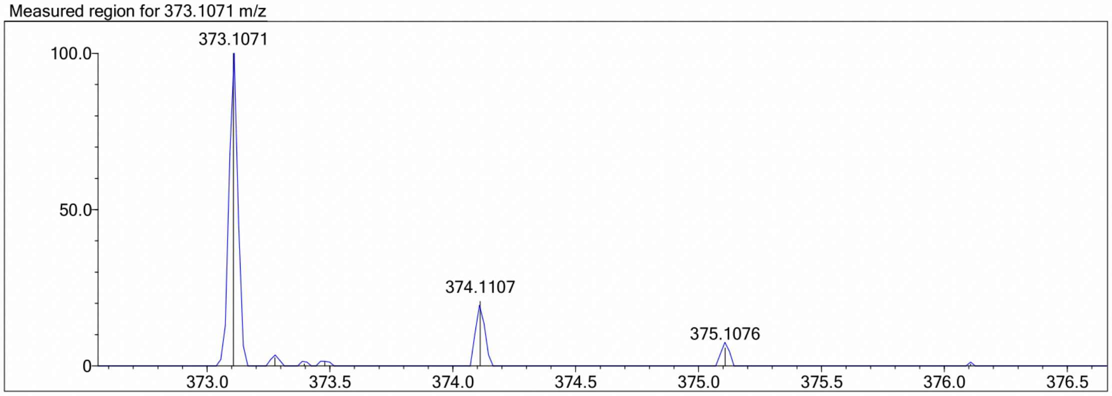


C16 H16 N6 O3 S [M+H]+ : Predicted region for 373.1077 m/z

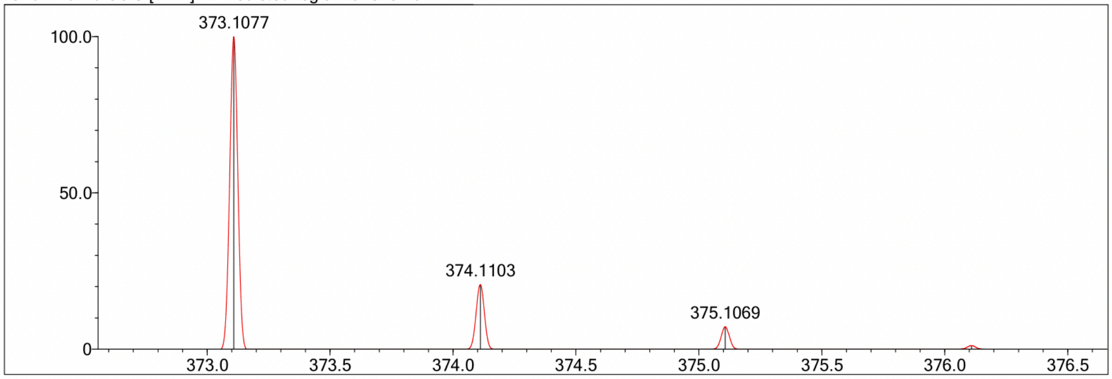


| Rank | Score | Formula (M) | Ion | Meas. m/z | Pred. m/z | Df. (mDa) | Df. (ppm) | Iso | DBE |
|---|---|---|---|---|---|---|---|---|---|
| 1 | 94.83 | C16 H16 N6 O3 S | [M+H]+ | 373.1071 | 373.1077 | -0.6 | -1.61 | 96.30 | 12.0 |

**Spectra 12.** HRMS spectra of compound **4d**

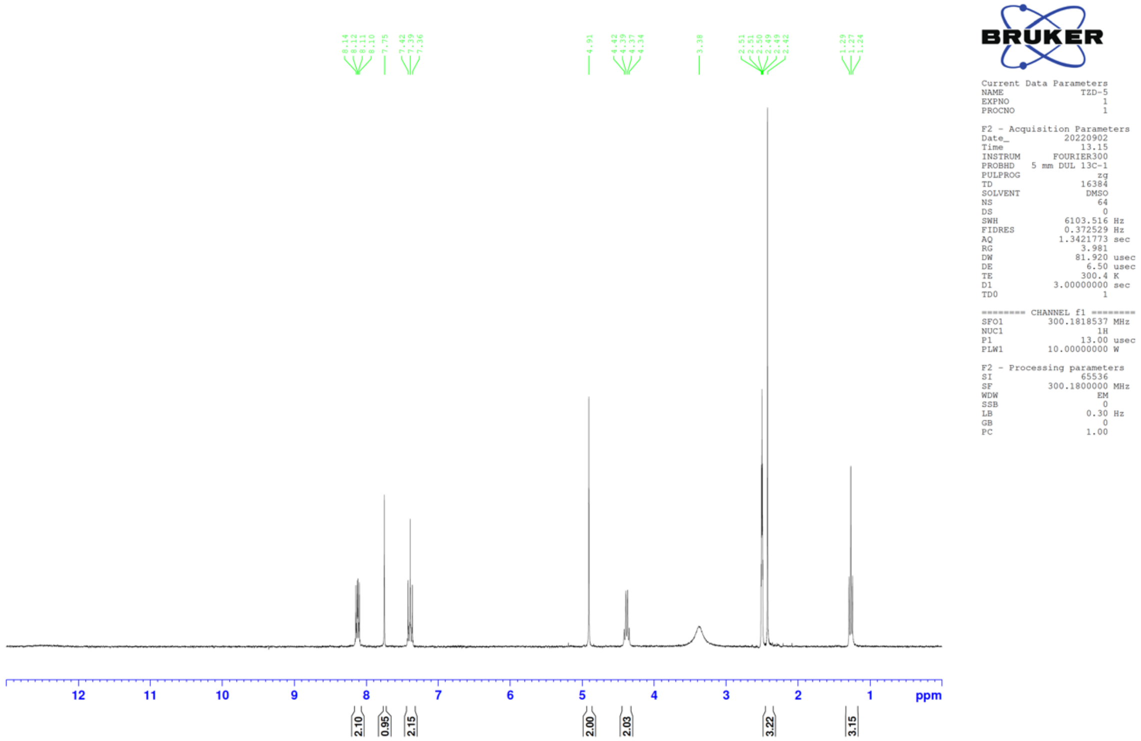


**Spectra 13.** $^{1}$H-NMR spectra of compound **4e**

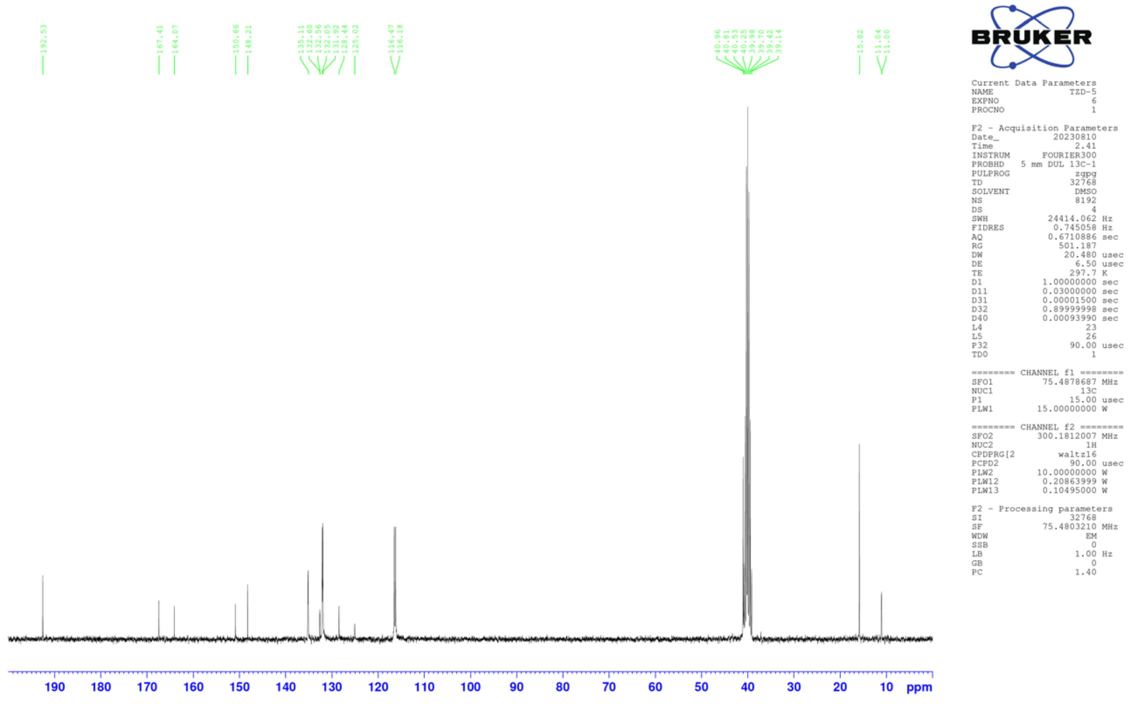


**Spectra 14.** $^{13}$C-NMR spectra of compound **4e**

Formula Predictor Report - T2D-5_238.lcd

Page 1 of 1

Data File: C:\LabSolutions\Data\Analiz\derya\T2D-5_238.lcd

| Elmt | Val. | Min | Max | Elmt | Val. | Min | Max | Elmt | Val. | Min | Max | Use Adduct |
|---|---|---|---|---|---|---|---|---|---|---|---|---|
| H | 1 | 16 | 35 | F | 1 | 1 | 1 | Br | 1 | 0 | 0 | H |
| C | 4 | 16 | 35 | P | 3 | 0 | 0 | Ru | 2 | 0 | 0 | Na |
| N | 3 | 5 | 6 | S | 2 | 1 | 1 | Pd | 2 | 0 | 0 | K |
| O | 2 | 0 | 3 | Cl | 1 | 0 | 0 | I | 3 | 0 | 0 | NH4 |

| | | | | | |
|---|---|---|---|---|---|
| Error Margin (ppm): | 5 | DBE Range: | 5.0 - 20.0 | Electron Ions: | both |
| HC Ratio: | unlimited | Apply N Rule: | yes | Use MSn Info: | yes |
| Max Isotopes: | 3 | Isotope RI (%): | 1.00 | Isotope Res: | 9000 |
| MSn Iso RI (%): | 10.00 | MSn Logic Mode: | AND | Max Results: | 50 |

Event#: 1 MS(E+) Ret. Time : 2.187 Scan# : 329

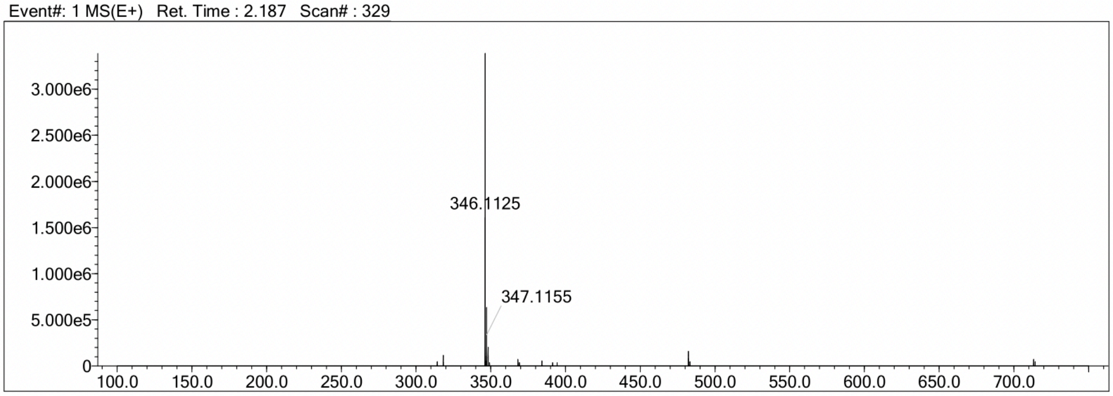


Measured region for 346.1125 m/z

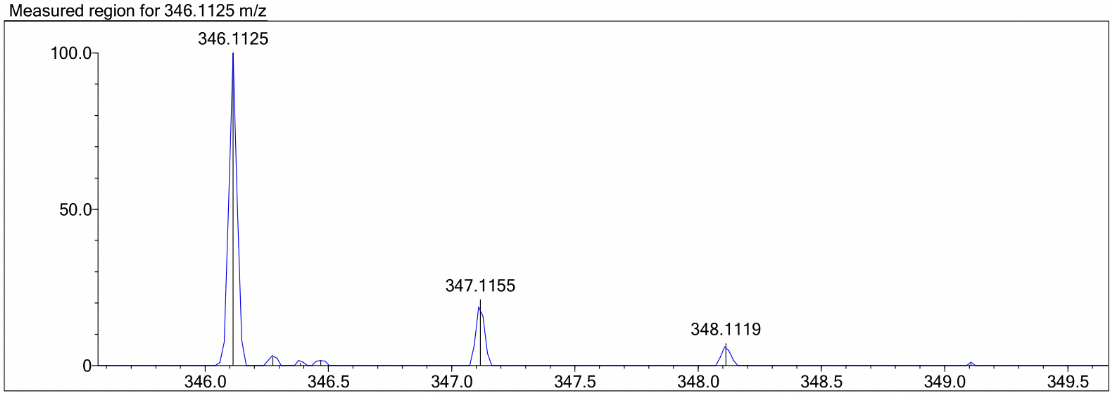


C16 H16 N5 O F S [M+H]+ : Predicted region for 346.1132 m/z

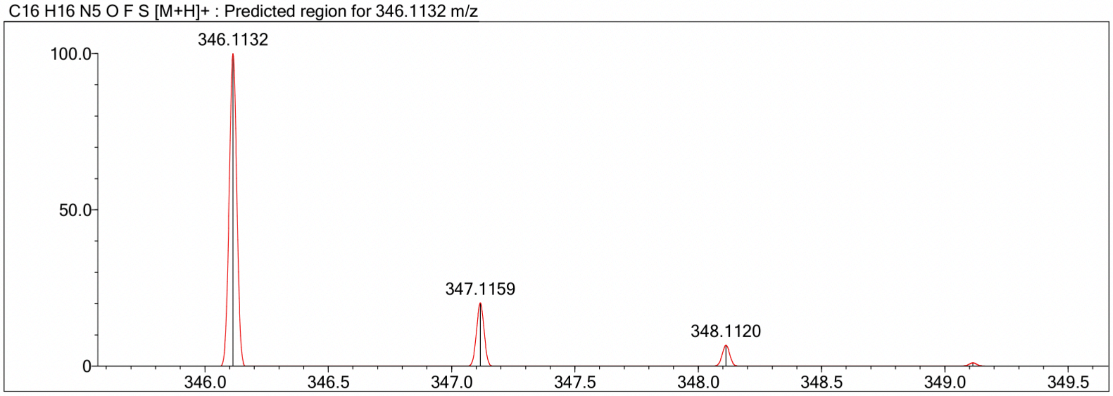


| Rank | Score | Formula (M) | Ion | Meas. m/z | Pred. m/z | Df. (mDa) | Df. (ppm) | Iso | DBE |
|---|---|---|---|---|---|---|---|---|---|
| 1 | 81.51 | C16 H16 N5 O F S | [M+H]+ | 346.1125 | 346.1132 | -0.7 | -2.02 | 83.64 | 11.0 |

**Spectra 15.** HRMS spectra of compound **4e**

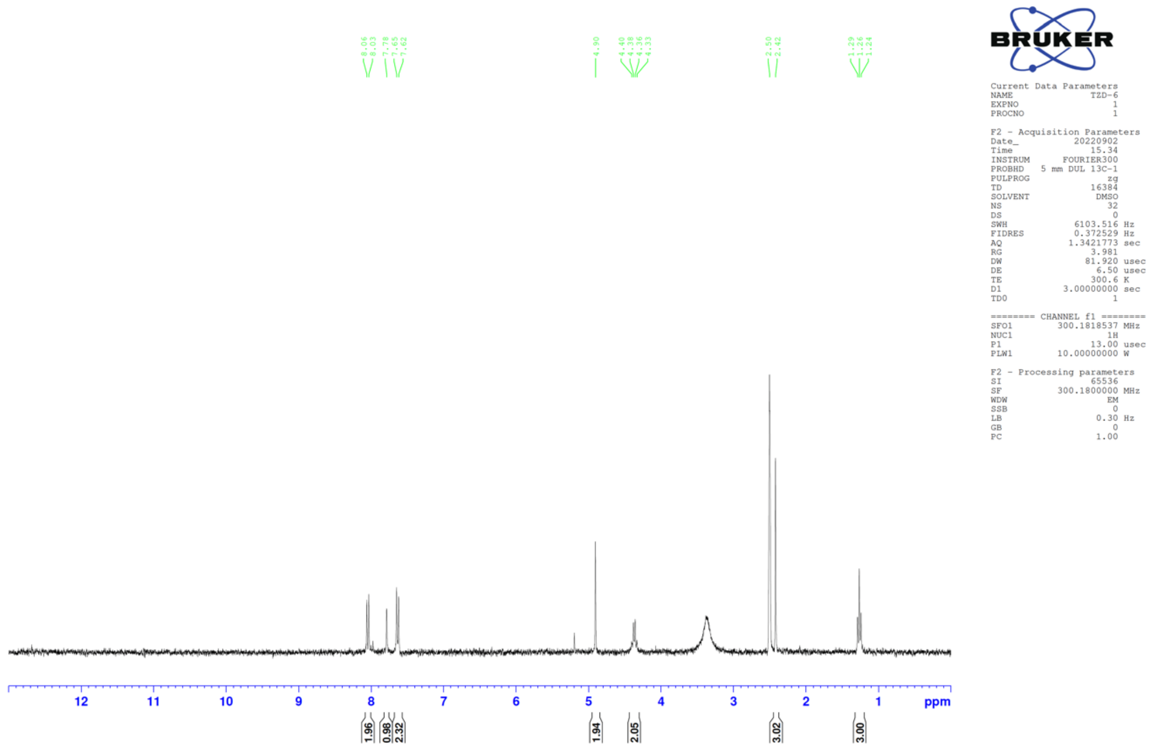

**Spectra 16.** $^{1}$H-NMR spectra of compound **4f**

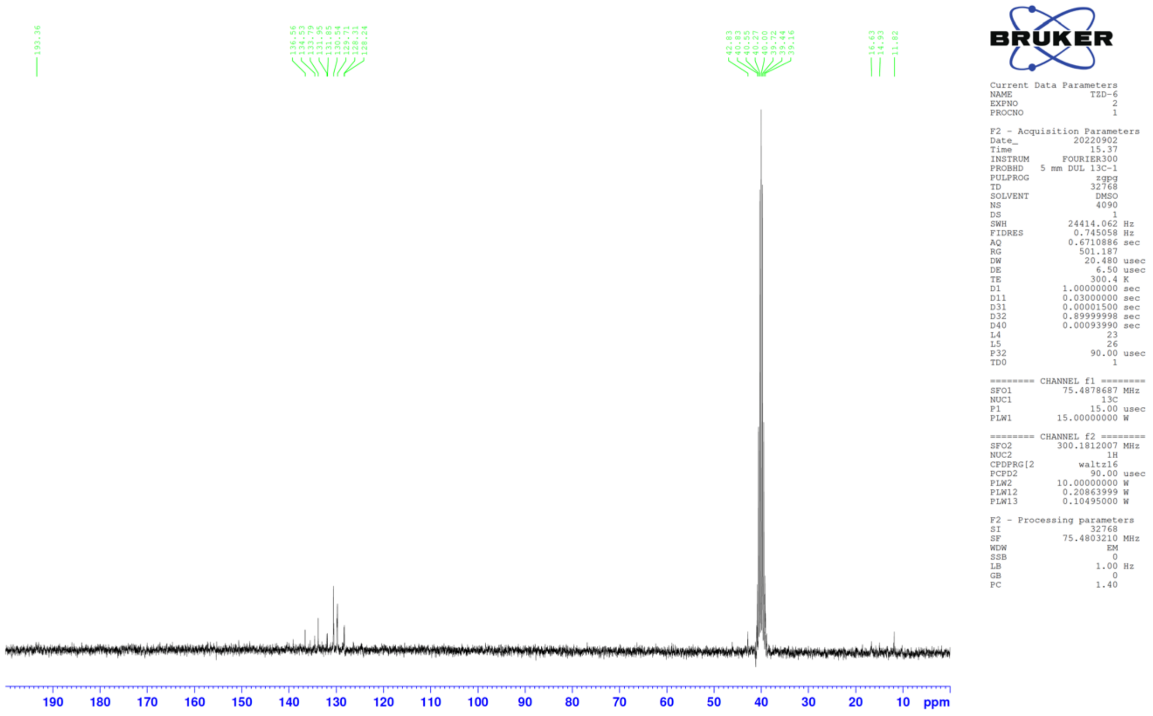

**Spectra 17.** $^{13}$C-NMR spectra of compound **4f**

Formula Predictor Report - T2D-6_239.lcd Page 1 of 1

Data File: C:\LabSolutions\Data\Analiz\derya\T2D-6_239.lcd

| Elmt | Val. | Min | Max | Elmt | Val. | Min | Max | Elmt | Val. | Min | Max | Use Adduct |
|---|---|---|---|---|---|---|---|---|---|---|---|---|
| H | 1 | 16 | 35 | F | 1 | 0 | 0 | Br | 1 | 0 | 0 | H |
| C | 4 | 16 | 35 | P | 3 | 0 | 0 | Ru | 2 | 0 | 0 | Na |
| N | 3 | 5 | 6 | S | 2 | 1 | 1 | Pd | 2 | 0 | 0 | K |
| O | 2 | 0 | 3 | Cl | 1 | 1 | 1 | I | 3 | 0 | 0 | NH4 |

| | | | | | |
|---|---|---|---|---|---|
| Error Margin (ppm): | 5 | DBE Range: | 5.0 - 20.0 | Electron Ions: | both |
| HC Ratio: | unlimited | Apply N Rule: | yes | Use MSn Info: | yes |
| Max Isotopes: | 3 | Isotope RI (%): | 1.00 | Isotope Res: | 9000 |
| MSn Iso RI (%): | 10.00 | MSn Logic Mode: | AND | Max Results: | 50 |

Event#: 1 MS(E+) Ret. Time : 2.320 Scan# : 349

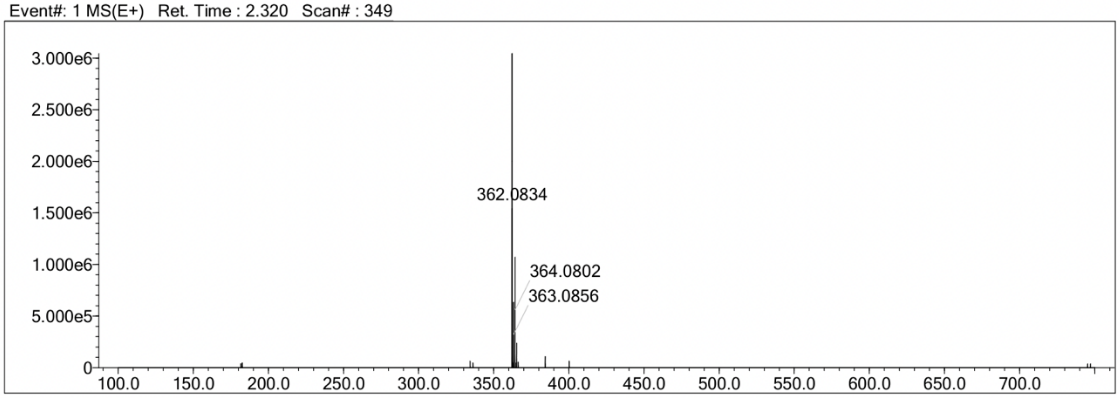


Measured region for 362.0834 m/z

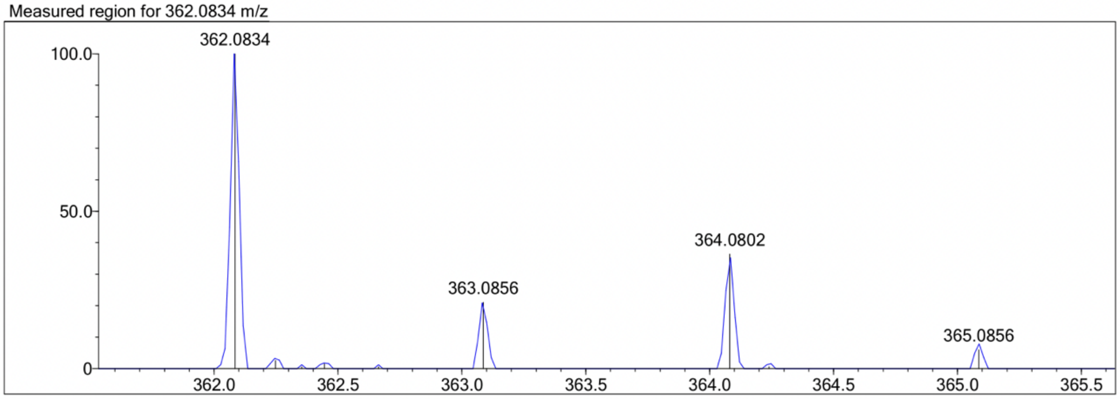


C16 H16 N5 O S Cl [M+H]+ : Predicted region for 362.0837 m/z

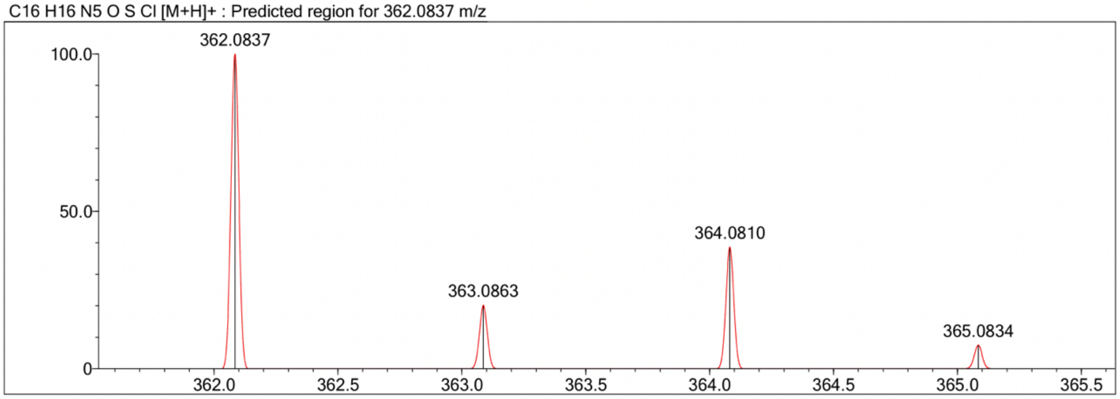


| Rank | Score | Formula (M) | Ion | Meas. m/z | Pred. m/z | Df. (mDa) | Df. (ppm) | Iso | DBE |
|---|---|---|---|---|---|---|---|---|---|
| 1 | 86.45 | C16 H16 N5 O S Cl | [M+H]+ | 362.0834 | 362.0837 | -0.3 | -0.83 | 86.45 | 11.0 |

**Spectra 18.** HRMS spectra of compound **4f**

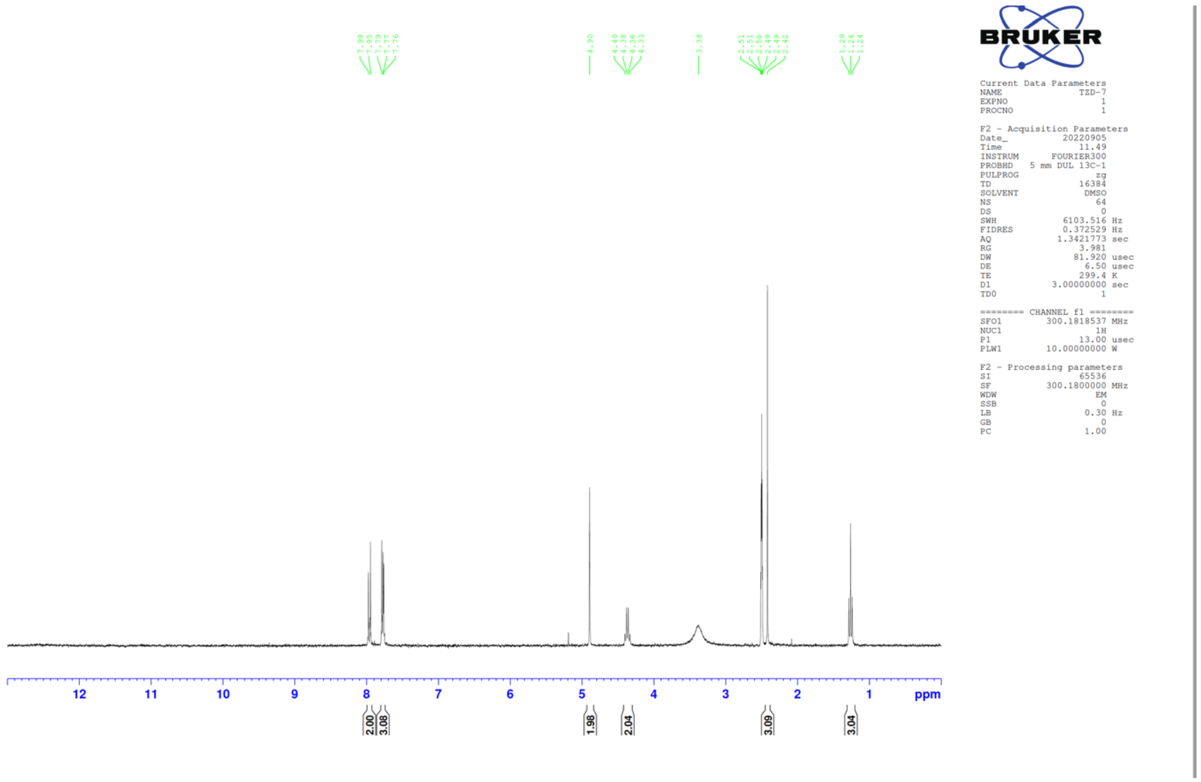


**Spectra 19.** $^{1}$H-NMR spectra of compound **4g**

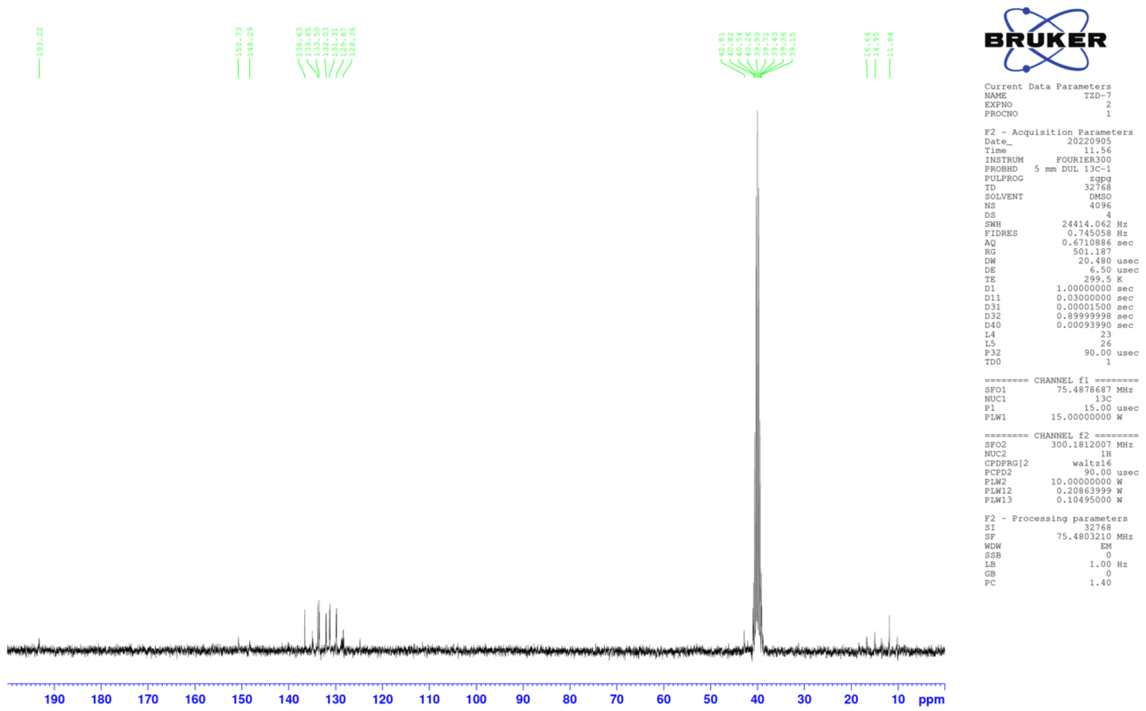


**Spectra 20.** $^{13}$C-NMR spectra of compound **4g**

Formula Predictor Report - T2D-7_240.lcd

Page 1 of 1

Data File: C:\LabSolutions\Data\Analiz\derya\T2D-7_240.lcd

| Elmt | Val. | Min | Max | Elmt | Val. | Min | Max | Elmt | Val. | Min | Max | Use Adduct |
|---|---|---|---|---|---|---|---|---|---|---|---|---|
| H | 1 | 16 | 35 | F | 1 | 0 | 0 | Br | 1 | 1 | 1 | H |
| C | 4 | 16 | 35 | P | 3 | 0 | 0 | Ru | 2 | 0 | 0 | Na |
| N | 3 | 5 | 6 | S | 2 | 1 | 1 | Pd | 2 | 0 | 0 | K |
| O | 2 | 0 | 3 | Cl | 1 | 0 | 0 | I | 3 | 0 | 0 | NH4 |

Error Margin (ppm): 5
HC Ratio: unlimited
Max Isotopes: 3
MSn Iso RI (%): 10.00

DBE Range: 5.0 - 20.0
Apply N Rule: yes
Isotope RI (%): 1.00
MSn Logic Mode: AND

Electron Ions: both
Use MSn Info: yes
Isotope Res: 9000
Max Results: 50

Event#: 1 MS(E+) Ret. Time : 2.307 Scan# : 347

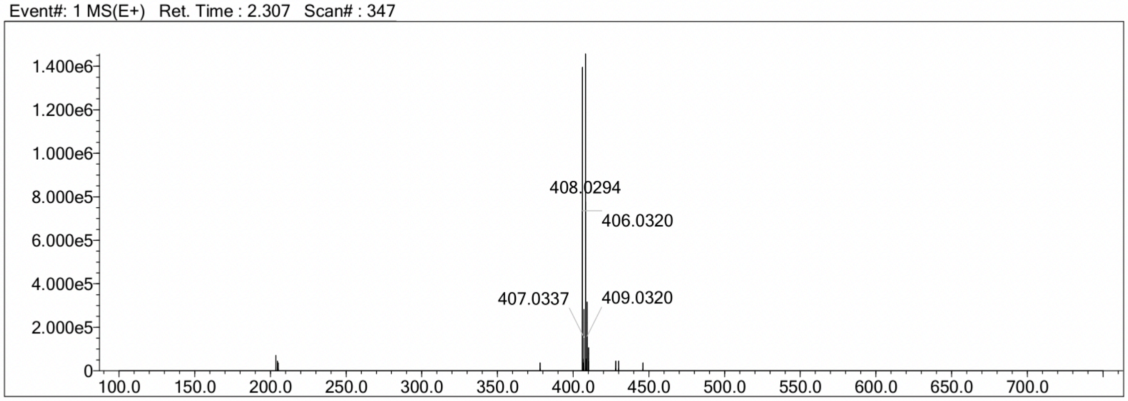


Measured region for 406.0320 m/z

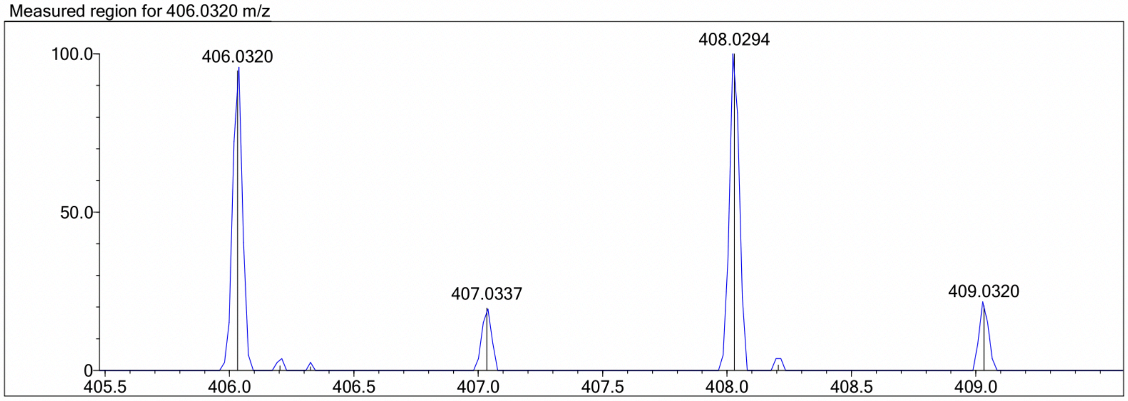


C16 H16 N5 O S Br [M+H]+ : Predicted region for 406.0332 m/z

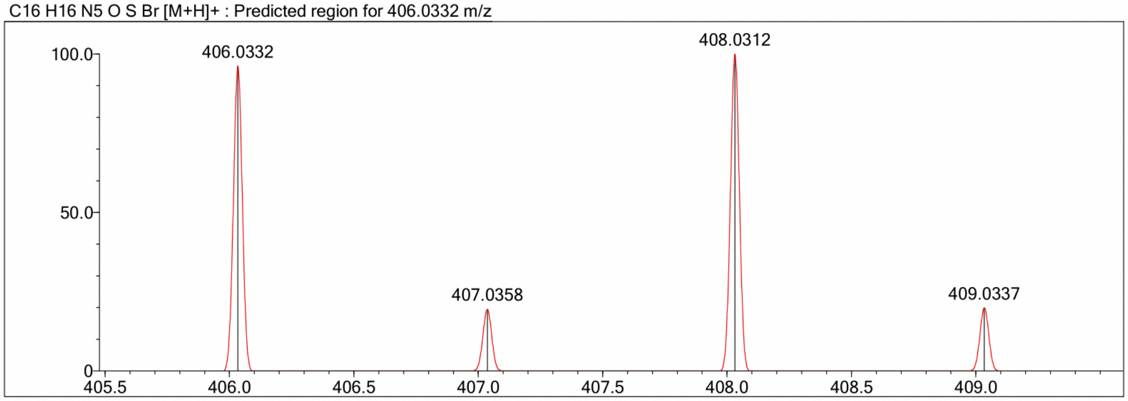


| Rank | Score | Formula (M) | Ion | Meas. m/z | Pred. m/z | Df. (mDa) | Df. (ppm) | Iso | DBE |
|---|---|---|---|---|---|---|---|---|---|
| 1 | 75.83 | C16 H16 N5 O S Br | [M+H]+ | 406.0320 | 406.0332 | -1.2 | -2.96 | 79.74 | 11.0 |

**Spectra 21.** HRMS spectra of compound **4g**

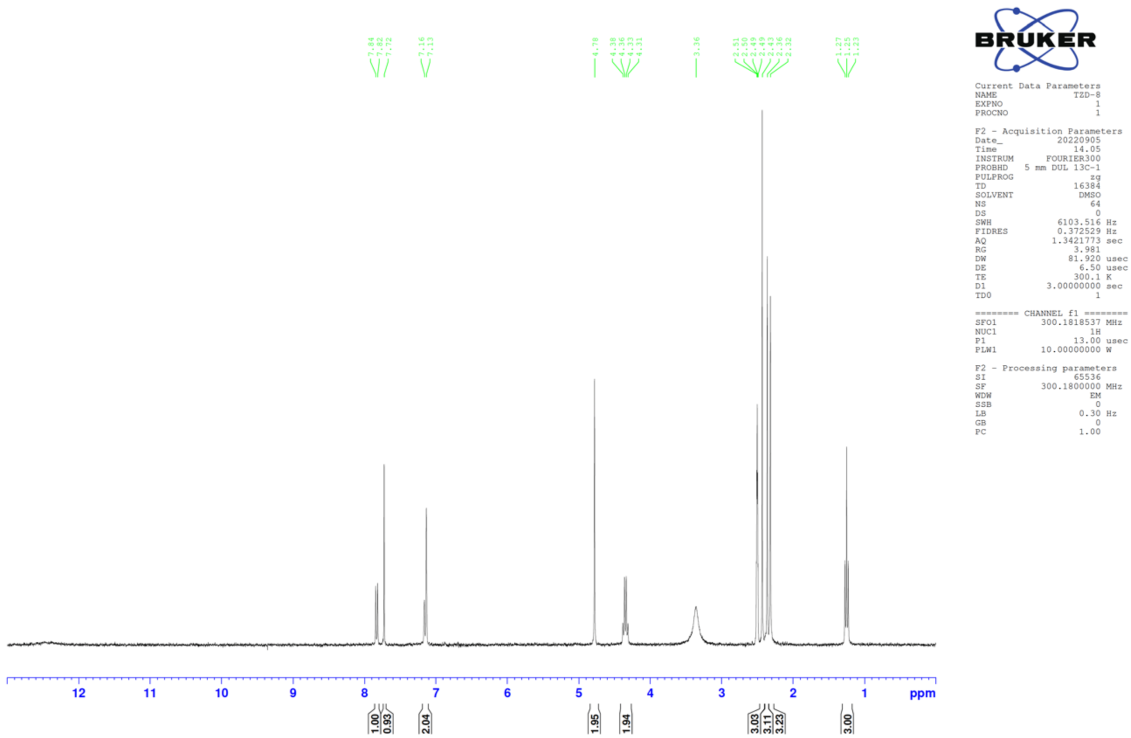


**Spectra 22.** $^{1}$H-NMR spectra of compound **4h**

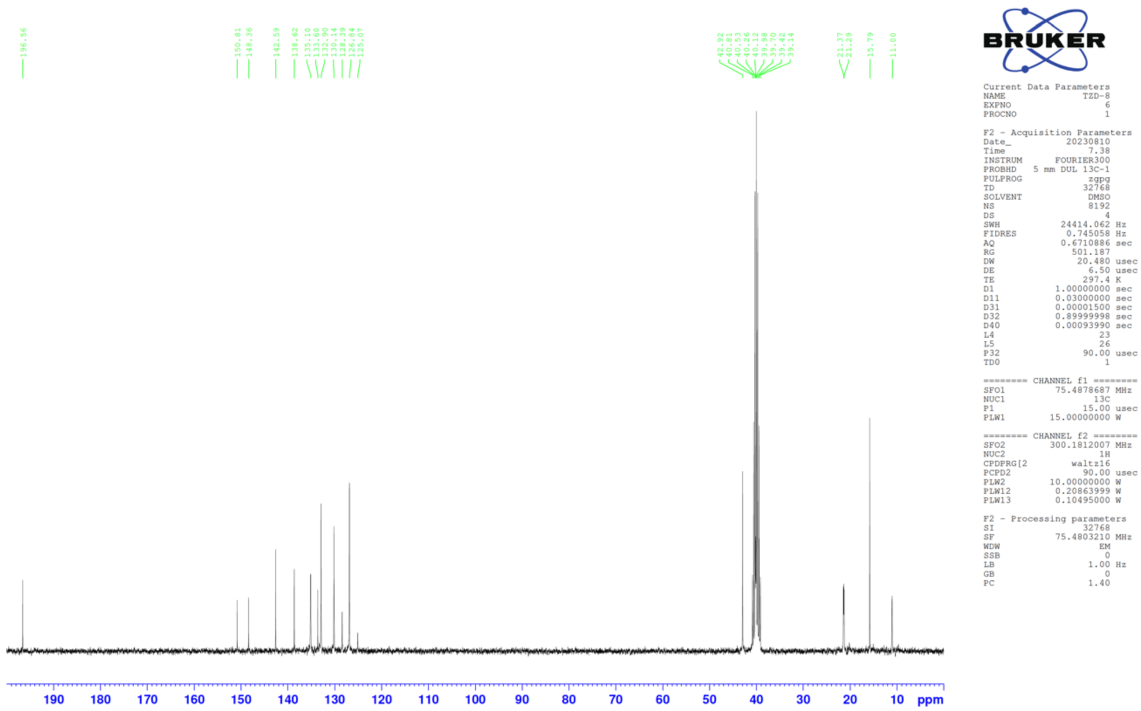


**Spectra 23.** $^{13}$C-NMR spectra of compound **4h**

Formula Predictor Report - T2D-8_241.lcd Page 1 of 1

Data File: C:\LabSolutions\Data\Analiz\derya\T2D-8_241.lcd

| Elmt | Val. | Min | Max | Elmt | Val. | Min | Max | Elmt | Val. | Min | Max | Use Adduct |
|---|---|---|---|---|---|---|---|---|---|---|---|---|
| H | 1 | 16 | 35 | F | 1 | 0 | 0 | Br | 1 | 0 | 0 | H |
| C | 4 | 16 | 35 | P | 3 | 0 | 0 | Ru | 2 | 0 | 0 | Na |
| N | 3 | 5 | 6 | S | 2 | 1 | 1 | Pd | 2 | 0 | 0 | K |
| O | 2 | 0 | 3 | Cl | 1 | 0 | 0 | I | 3 | 0 | 0 | NH4 |

| | | | | | |
|---|---|---|---|---|---|
| Error Margin (ppm): | 5 | DBE Range: | 5.0 - 20.0 | Electron Ions: | both |
| HC Ratio: | unlimited | Apply N Rule: | yes | Use MSn Info: | yes |
| Max Isotopes: | 3 | Isotope RI (%): | 1.00 | Isotope Res: | 9000 |
| MSn Iso RI (%): | 10.00 | MSn Logic Mode: | AND | Max Results: | 50 |

Event#: 1 MS(E+) Ret. Time : 2.547 Scan# : 383

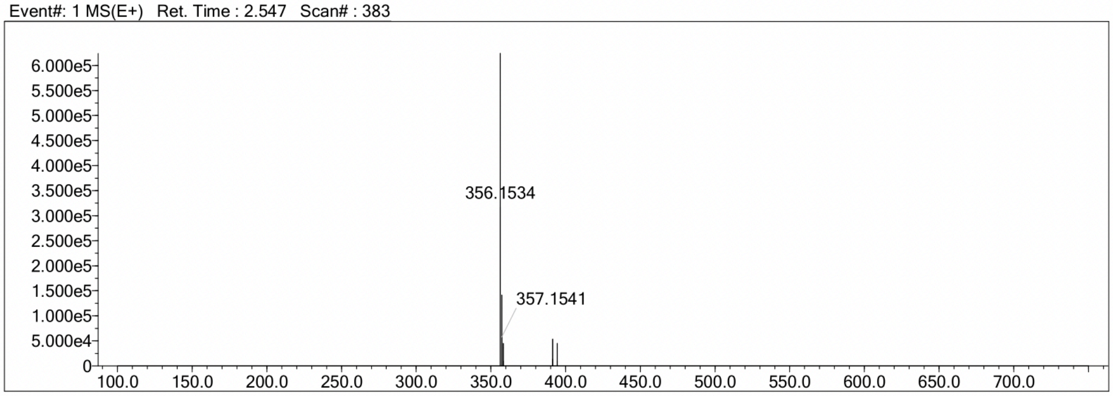


Measured region for 356.1534 m/z

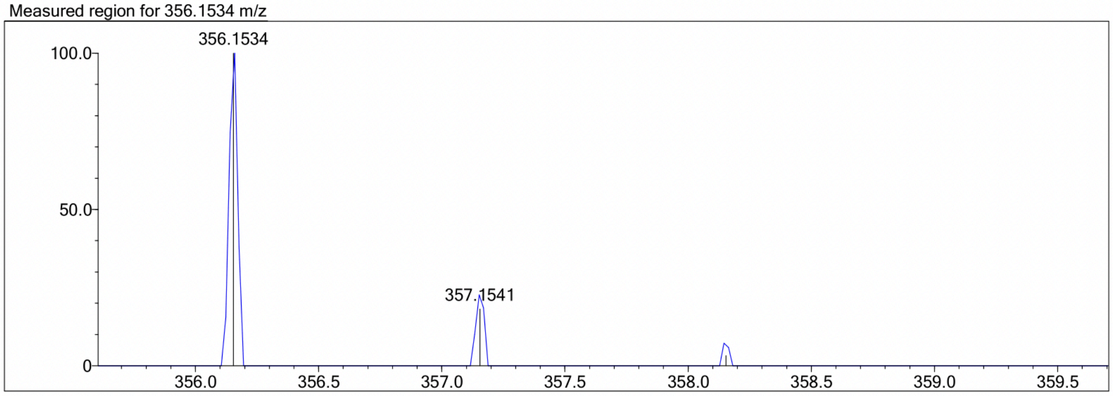


C18 H21 N5 O S [M+H]+ : Predicted region for 356.1540 m/z

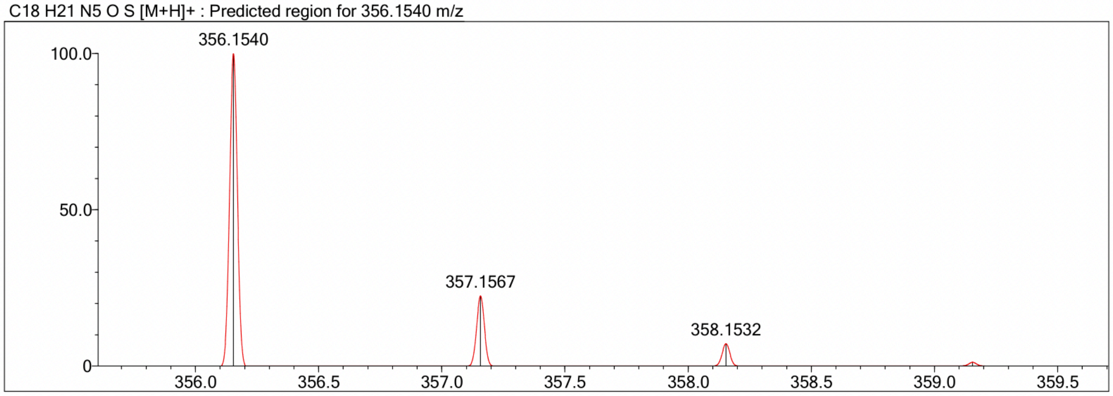


| Rank | Score | Formula (M) | Ion | Meas. m/z | Pred. m/z | Df. (mDa) | Df. (ppm) | Iso | DBE |
|---|---|---|---|---|---|---|---|---|---|
| 1 | 73.96 | C18 H21 N5 O S | [M+H]+ | 356.1534 | 356.1540 | -0.6 | -1.68 | 75.24 | 11.0 |

**Spectra 24.** HRMS spectra of compound **4h**

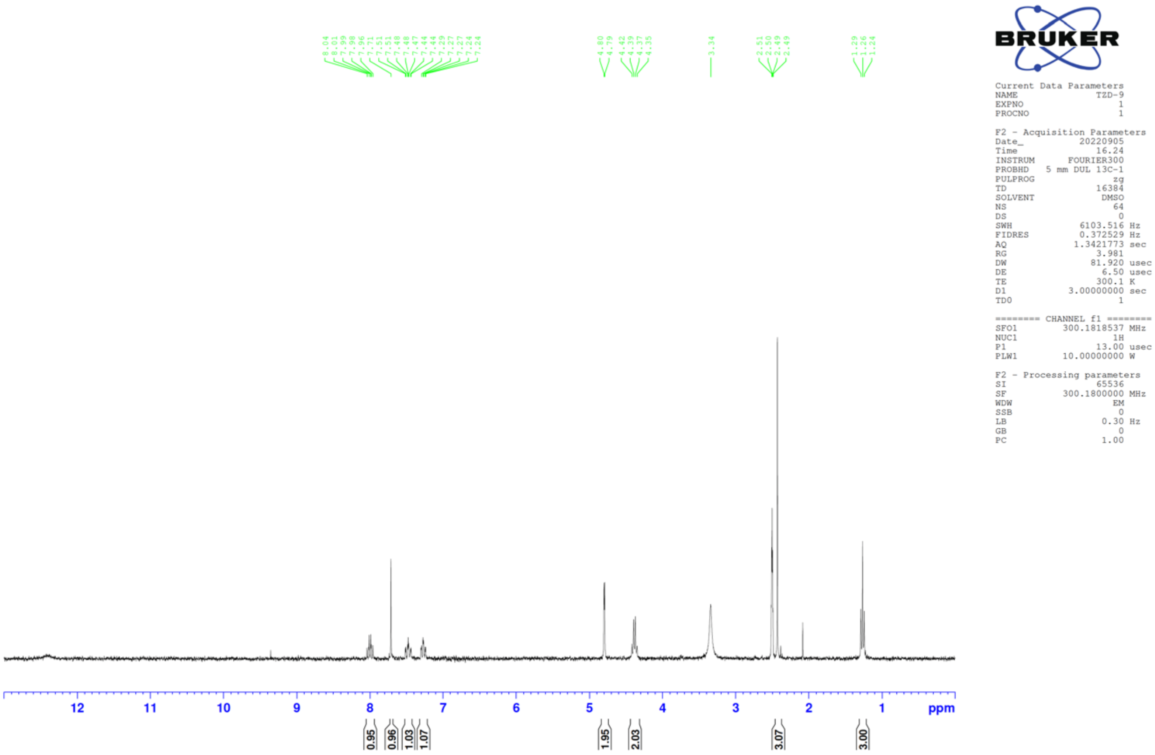


**Spectra 25.** $^{1}$H-NMR spectra of compound **4i**

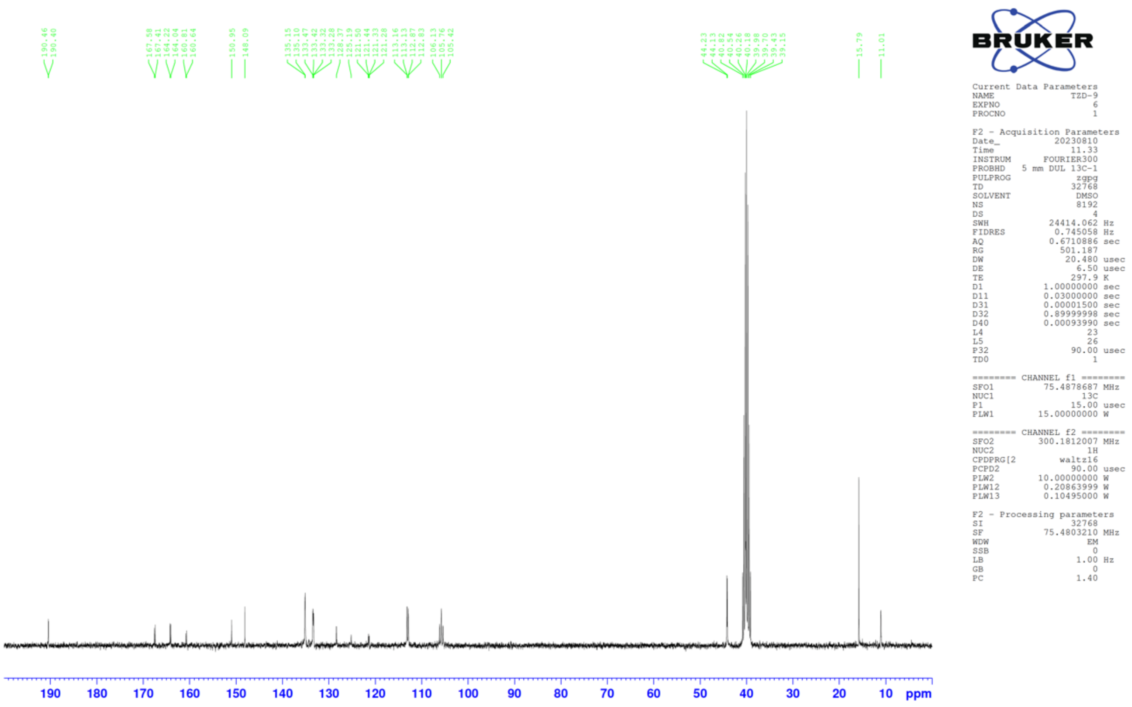


**Spectra 26.** $^{13}$C-NMR spectra of compound **4i**

Formula Predictor Report - T2D-10_243.lcd Page 1 of 1

Data File: C:\LabSolutions\Data\Analiz\derya\T2D-10_243.lcd

| Elmt | Val. | Min | Max | Elmt | Val. | Min | Max | Elmt | Val. | Min | Max | Use Adduct |
|---|---|---|---|---|---|---|---|---|---|---|---|---|
| H | 1 | 15 | 35 | F | 1 | 2 | 2 | Br | 1 | 0 | 0 | H |
| C | 4 | 16 | 35 | P | 3 | 0 | 0 | Ru | 2 | 0 | 0 | Na |
| N | 3 | 5 | 6 | S | 2 | 1 | 1 | Pd | 2 | 0 | 0 | K |
| O | 2 | 0 | 3 | Cl | 1 | 0 | 0 | I | 3 | 0 | 0 | NH4 |

| | | | | | |
|---|---|---|---|---|---|
| Error Margin (ppm): | 5 | DBE Range: | 5.0 - 20.0 | Electron Ions: | both |
| HC Ratio: | unlimited | Apply N Rule: | yes | Use MSn Info: | yes |
| Max Isotopes: | 3 | Isotope RI (%): | 1.00 | Isotope Res: | 9000 |
| MSn Iso RI (%): | 10.00 | MSn Logic Mode: | AND | Max Results: | 50 |

Event#: 1 MS(E+) Ret. Time : 2.067 Scan# : 311

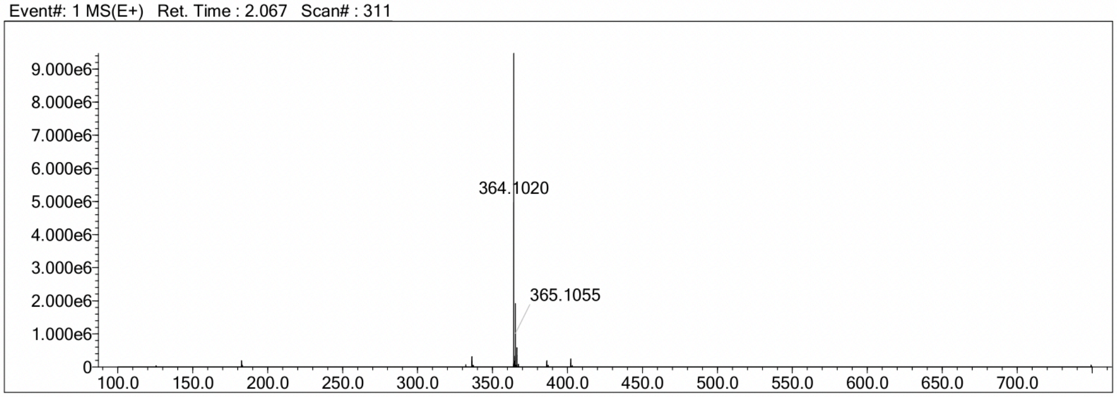


Measured region for 364.1020 m/z

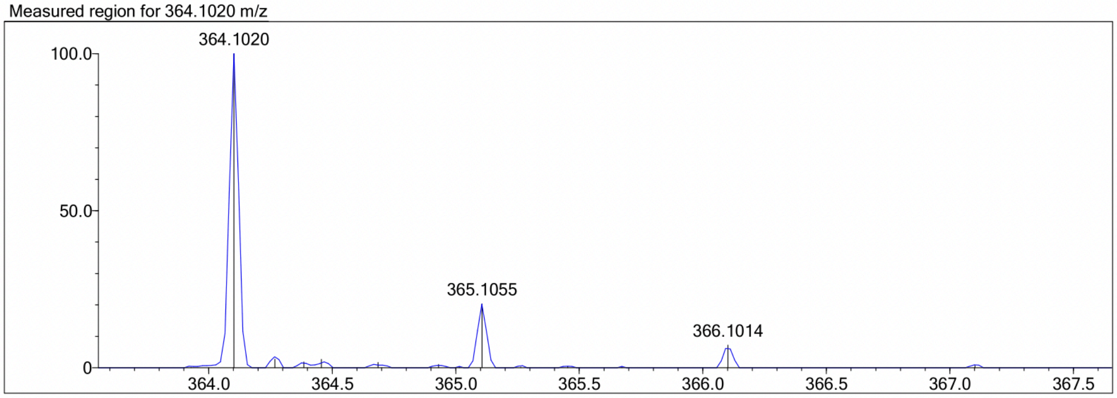


C16 H15 N5 O F2 S [M+H]+ : Predicted region for 364.1038 m/z

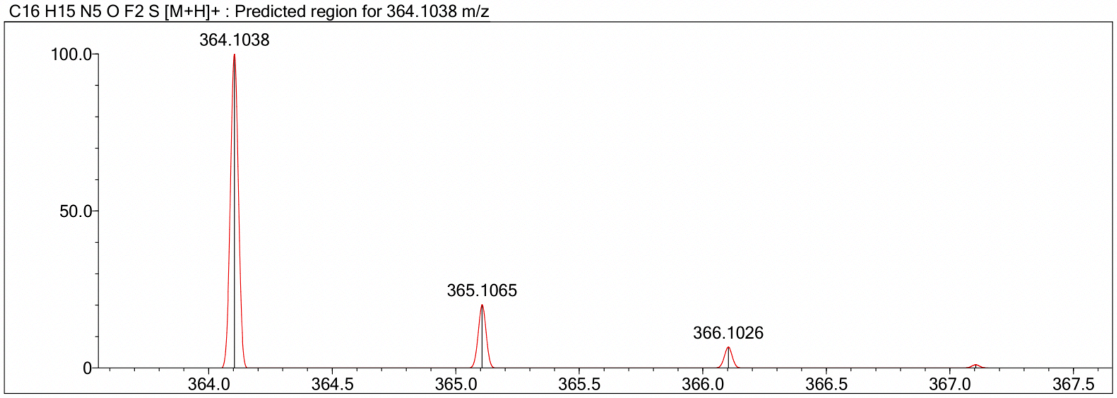


| Rank | Score | Formula (M) | Ion | Meas. m/z | Pred. m/z | Df. (mDa) | Df. (ppm) | Iso | DBE |
|---|---|---|---|---|---|---|---|---|---|
| 1 | 88.21 | C16 H15 N5 O F2 S | [M+H]+ | 364.1020 | 364.1038 | -1.8 | -4.94 | 97.85 | 11.0 |

**Spectra 27.** HRMS spectra of compound **4i**

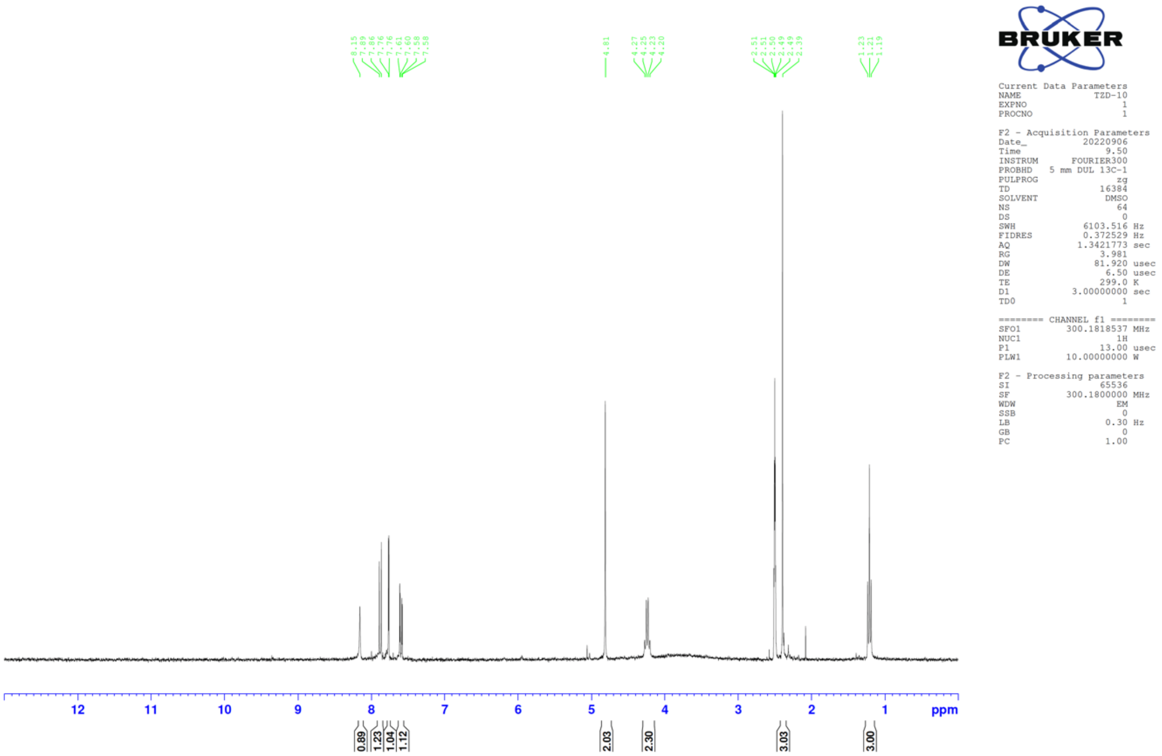


**Spectra 28.** $^{1}$H-NMR spectra of compound **4j**

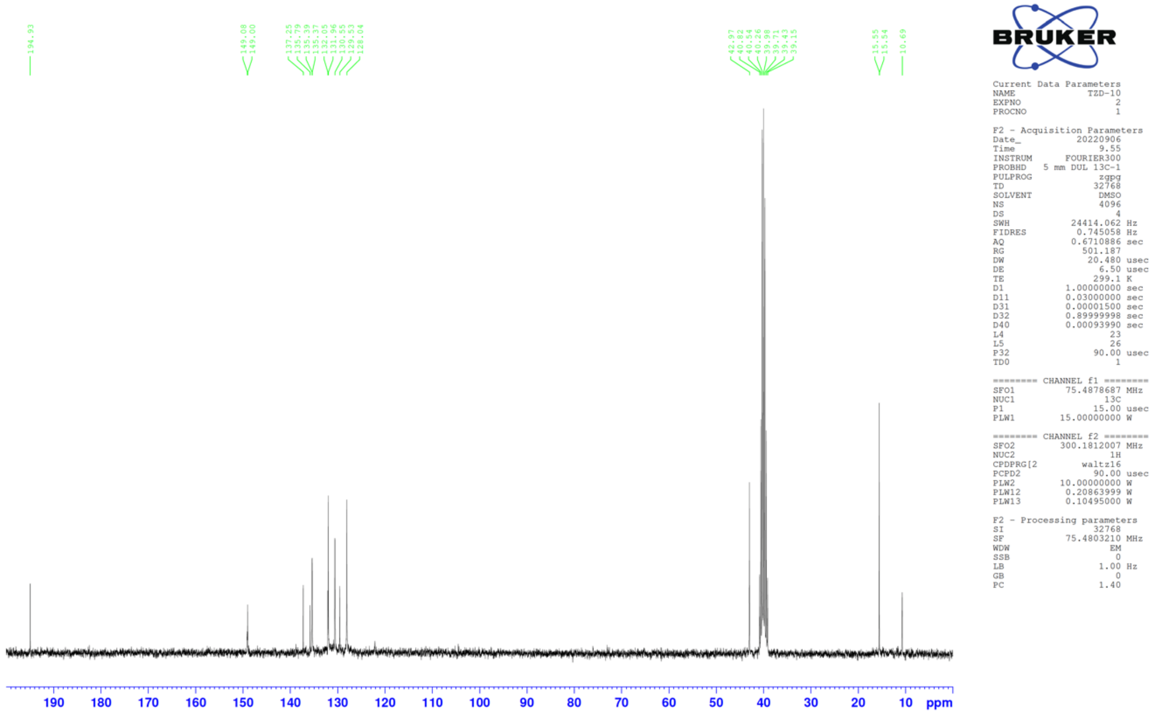


**Spectra 29.** $^{13}$C-NMR spectra of compound **4j**

Formula Predictor Report - T2D-9_242.lcd Page 1 of 1

Data File: C:\LabSolutions\Data\Analiz\derya\T2D-9_242.lcd

| Elmt | Val. | Min | Max | Elmt | Val. | Min | Max | Elmt | Val. | Min | Max | Use Adduct |
|---|---|---|---|---|---|---|---|---|---|---|---|---|
| H | 1 | 15 | 35 | F | 1 | 0 | 0 | Br | 1 | 0 | 0 | H |
| C | 4 | 16 | 35 | P | 3 | 0 | 0 | Ru | 2 | 0 | 0 | Na |
| N | 3 | 5 | 6 | S | 2 | 1 | 1 | Pd | 2 | 0 | 0 | K |
| O | 2 | 0 | 3 | Cl | 1 | 2 | 2 | I | 3 | 0 | 0 | NH4 |

| | | | | | |
|---|---|---|---|---|---|
| Error Margin (ppm): | 5 | DBE Range: | 5.0 - 20.0 | Electron Ions: | both |
| HC Ratio: | unlimited | Apply N Rule: | yes | Use MSn Info: | yes |
| Max Isotopes: | 3 | Isotope RI (%): | 1.00 | Isotope Res: | 9000 |
| MSn Iso RI (%): | 10.00 | MSn Logic Mode: | AND | Max Results: | 50 |

Event#: 1 MS(E+) Ret. Time : 2.267 Scan# : 341

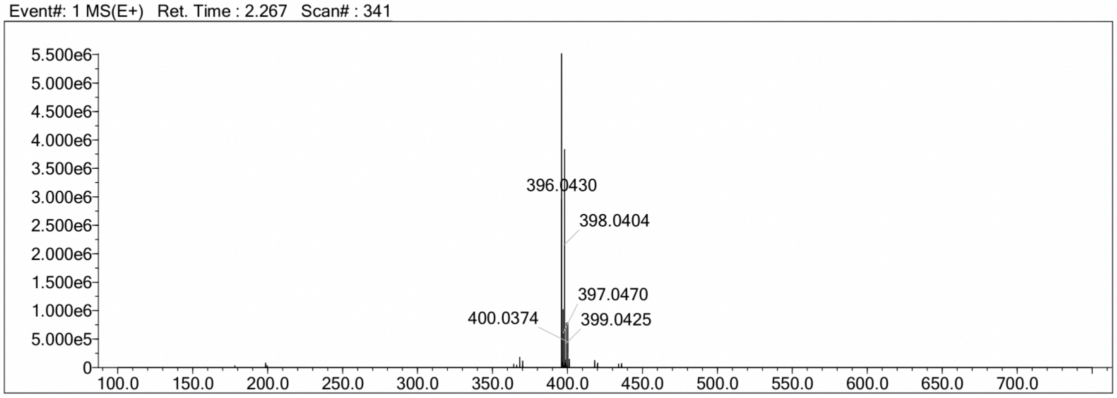


Measured region for 396.0430 m/z

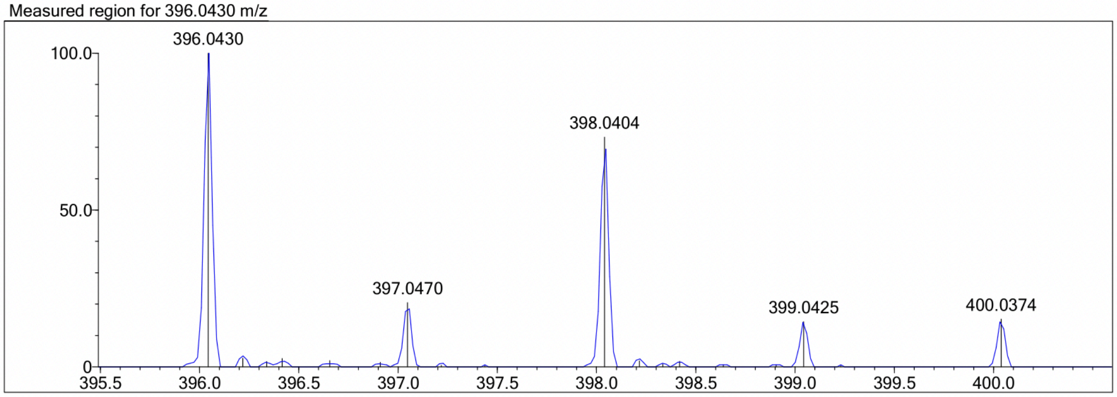


C16 H15 N5 O S Cl2 [M+H]+ : Predicted region for 396.0447 m/z

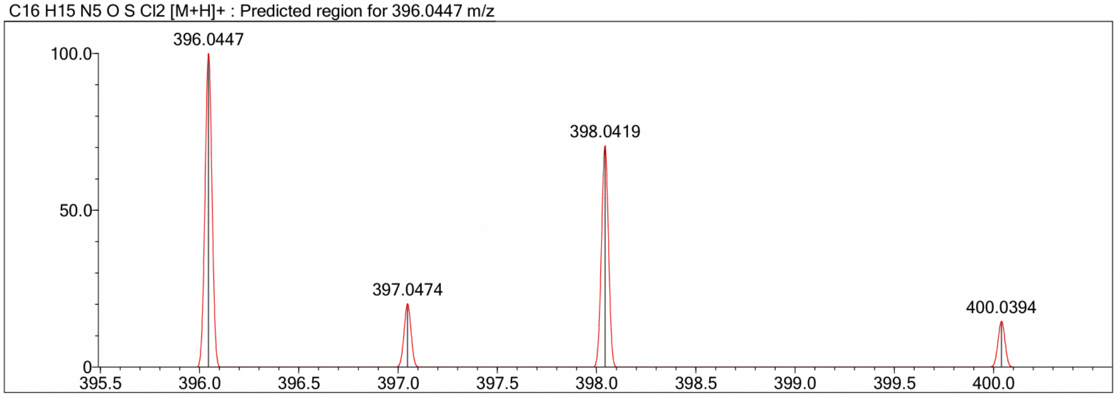


| Rank | Score | Formula (M) | Ion | Meas. m/z | Pred. m/z | Df. (mDa) | Df. (ppm) | Iso | DBE |
|---|---|---|---|---|---|---|---|---|---|
| 1 | 84.90 | C16 H15 N5 O S Cl2 | [M+H]+ | 396.0430 | 396.0447 | -1.7 | -4.29 | 92.51 | 11.0 |

**Spectra 30.** HRMS spectra of compound **4j**
